# Concatenation, Splicing and Insertion–Deletion Operations on Cyclic DNA Molecules

**DOI:** 10.3390/e28101113

**Published:** 2026-10-12

**Authors:** Bilal Abdallah, Benedek Nagy

**Affiliations:** 1 Department of Mathematics, Faculty of Arts and Sciences, Eastern Mediterranean University, via Mersin-10, 99450 Famagusta, North Cyprus, Türkiye; 2 College of Business Administration, American University of the Middle East, Egaila 54200, Kuwait; 3 Department of Computer Science, Institute of Mathematics and Informatics, Eszterházy Károly Catholic University, 3300 Eger, Hungary

**Keywords:** circular words, splicing systems, H-systems, insertion–deletion systems, DNA operations, concatenation of necklaces, insertion–deletion to circular words, circular splicing, biological information processing

## Abstract

Splicing systems (H-systems) and insertion-deletion systems are well-studied DNA computing paradigms inspired by biological operations observed in nature. They are modelled in a formal, information-theoretic manner. In these systems, splicing, insertion and deletion operations are used. In this paper, we study these operations on circular words, which naturally model cyclic DNA molecules, focusing on the relationship between algebraic operations and biologically inspired string transformations. In particular, we formally compare concatenation with circular splicing and insertion. While the splicing operation has already been defined and used for circular words in the literature, for insertion–deletion systems, e.g., circular insertion was defined to insert a linearized variant of a circular string (cyclic molecule) into a linear (ordinary) string (or linear molecule that can be represented by a string). In this paper, we provide a formal definition of insertion operators acting on circular words. We prove that if we use circular splicing and insertion in a somewhat context-independent way (using the empty word in the formal rules), these computational operations coincide with the one-wheel concatenation operation. Our results support the theoretical understanding of these operations and their biological interpretations, with implications for DNA computing and synthetic biology.

## 1. Introduction

In the field of molecular chemistry and biology, chemists have worked for centuries to obtain ever newer molecules which play essential roles in complex biological systems and are useful in medicine or in industrial applications [1]. Thinking about DNA molecules as important biological molecules, there are various enzymes that are able to modify them, and these enzymes can be used to build them or to make some changes to them [2,3]. A branch of computing and information theory, also related to formal language theory, is used to provide formal models of these operations. In this paper, we concentrate on modeling circular (DNA) molecules and computational operations on them.

Circular splicing is a well-explored concept in both molecular biology and theoretical computer science, primarily due to the significance of circular DNA in various biological processes and the insight splicing systems provide into the properties of circular words [4,5]. Despite extensive investigation, many classical problems related to circular splicing—such as characterizing all circular languages generated by finite circular splicing systems and understanding their computational power—remain unsolved in formal language theory [6,7].

The splicing operation was first proposed in 1987 by Tom Head [8]. Formal systems based on splicing operations are called splicing systems or H-systems. Further, he also introduced circular splicing systems in 1992 [9], along with a number of open problems concerning their computational capabilities. Then the idea was generalized by Păun [10,11] and Pixton [12] in 1996 to simulate the recombinant behavior of DNA sequences. The splicing concept is inspired by the action of two enzymes that cut DNA molecules (represented by strings here) at designated substrings, referred to as sites, and then reattach the first segment of one string to the second segment of the other, appropriately modifying the sites. From a mathematical and information-theoretical modeling perspective, we express this in the following way:

Let Σ be an arbitrary finite alphabet. A splicing rule over Σ is defined as a string of the form *u*_1_#*u*_2_$*u*_3_#*u*_4_ where *u*_1_, *u*_2_, *u*_3_, *u*_4_ are strings over Σ while # and $ are special symbols not contained in Σ. Intuitively, the pairs (*u*_1_,*u*_2_) and (*u*_3_,*u*_4_) model the recognition sites of two restriction enzymes; the first enzyme cuts the first string (molecule) between *u*_1_ and *u*_2_, while the second enzyme cuts the second between *u*_3_ and *u*_4_. To shorten the notation, we will represent the splicing rules by quadruples of the form (*u*_1_, *u*_2_; *u*_3_, *u*_4_). For a given splicing rule *r* = (*u*_1_, *u*_2_; *u*_3_, *u*_4_) and three strings *x*, *y*, *z* over Σ, we denote the relation (*x*,*y*)⊢*_r_z* if the following conditions hold: *x* = *x*_1_*u*_1_*u*_2_*x*_2_, *y* = *y*_1_*u*_3_*u*_4_*y*_2_, *z* = *x*_1_*u*_1_*u*_4_*y*_2_ for some *x*_1_, *x*_2_, *y*_1_, *y*_2_ ∈ Σ^*^. We may also examine the symmetric rule *r′* = (*u*_3_, *u*_4_; *u*_1_, *u*_2_), which yields the result *w* = *y*_1_*u*_3_*u*_2_*x*_2_ where (*y*,*x*)⊢*_r′_w*. We will recall the circular splicing operation at the beginning of Section 3.

Similar to splicing, the insertion and deletion operations have gained attention as abstract models of bio-molecular processes. Moreover, they also have a central role in formal language theory and in formal models of DNA computing [13]. These operations reflect fundamental mechanisms such as the insertion and removal of DNA segments, and thus provide a natural basis for bio-inspired computational models [11].

The insertion and deletion operations were introduced for linear words [14]. Their circular variants were studied in [15], where a circular word is inserted into a linear word: the cyclic molecule is cut, and its linearization is placed between two contexts of a linear host molecule. Thus, in the known formulations, only the inserted molecule is cyclic, while the host, and hence also the result, is linear. What has not been defined so far is the operation in which the host molecule is cyclic as well, that is, an insertion that takes two circular words and returns a circular word, and, symmetrically, a deletion that removes a cyclic factor from a circular word. This is precisely the situation modeled in this paper. The gap is not merely notational: an operation whose result is linear cannot be iterated within a family of circular languages, nor can it be compared with circular splicing operations or the concatenation of circular words, as all of these operations act on circular words. Moreover, as we show in Section 4, applying the known operation to a linearization of a circular word and circularizing the outcome does not give the same result, because the occurrences of the context that span the position at which the circular word was cut are not exhibited by any linear representative of the molecule.

To define the insertion–deletion operations, we consider Σ as a finite alphabet, and *R* is a finite set of triples of the form (*u*,*α*/*β*,*v*) where *u*, *v* ∈Σ^*^, (*α*,*β*) ∈ (Σ^+^×{*λ*}) ∪ ({*λ*} × Σ^+^). The triples in *R* are called insertion–deletion rules, and they are used as follows: *r_ins_* = (*u*,*λ*/*β*,*v*) means that *β* can be inserted between *u* and *v* in accordance with the rewriting rule *uv* → *uβv*, while *r_del_* = (*u*,*α*/*λ*,*v*) means that *α* can be deleted from the (*u*,*v*) context corresponding to the rewriting rule *uαv* → *uv*. Thus, for *x*,*y* ∈ Σ^*^, we denote the relations $x{⊢}_{r_{\mathrm{ins}}}y$ and $x{⊢}_{r_{\mathrm{del}}}y$ if *y* can be obtained from *x* by using an insertion and deletion rule, respectively, as defined above. In other words, one of the following two cases holds:

- *x* = *x*_1_*uvx*_2_, *y* = *x*_1_*uβvx*_2_ for *x*_1_, *x*_2_ ∈ Σ^*^, *β* ∈ Σ^+^ and *r_ins_* = (*u*,*λ*/*β*,*v*) ∈ *R*;
- *x* = *x*_1_*uαvx*_2_, *y* = *x*_1_*uvx*_2_ for *x*_1_, *x*_2_ ∈ Σ^*^, *α* ∈ Σ^+^ and *r_del_* = (*u*,*α*/*λ*,*v*) ∈ *R*.

Note that (*α*,*β*) ≠ (*λ*,*λ*) by definition, as no insertion or deletion occurs in this case. The circular extension of the insertion and deletion operations will be defined formally at the beginning of Section 4.

The concatenation operation is the usual way to unite information represented by words: it makes a new word from the two input words by continuing the first word, after its last letter, with the first letter of the second word. However, circular words have neither first nor last letters (see the formal descriptions in the next section); thus, it is not straightforward to generalize concatenation for them. Therefore, there are various concatenation operators defined for circular words [16]. In [17], two concatenation operations were considered, namely, the one-wheel and two-wheel concatenations. Their naming is motivated by the graphical representation of a circular word as a wheel, in which its letters are arranged cyclically around a circle. Under this interpretation, one-wheel concatenation combines two circular words into a single wheel, and thus the result consists of circular words, whereas two-wheel concatenation retains the two circular words as two distinct wheels while establishing the concatenation between them, and therefore may produce more complex structures.

This paper explores the theoretical foundations of circular splicing and insertion-deletion operations used in the corresponding modeling systems, with a focus on their computational power and biological relevance. We will examine various models, their relations to the one-wheel concatenation [17] and the open questions that continue to challenge researchers in this field.

The main contributions of this paper are the following:

- We prove that Head’s and Păun’s circular splicing operations produce the same circular words under an explicit translation of the rules, i.e., as single operations they are equivalent in the sense that one operation can be simulated by an operation of the other type (Proposition 1);
- We prove that the one-wheel concatenation of two circular words is exactly the set obtained by all applicable Păun circular splicing rules, and also by the single rule (*λ*, *λ*; *λ*, *λ*), and the analogous result for Pixton’s operation with the rule pair (*λ*, *λ*; *λ*) (Theorem 2, Corollary 1 and Theorem 4);
- We give if-and-only-if characterizations of the cases when the Păun and the Pixton circular splicing operations coincide with the two-wheel concatenation (Theorems 3 and 5);
- We provide a formal definition of the insertion operation acting on circular words, establish the hierarchy of the obtained sets and characterize when they coincide, and prove that this insertion can be simulated by Păun circular splicing and that, with empty contexts, it coincides with the one-wheel concatenation (Definition 7, Lemmas 2 and 3, Theorems 6–8);
- We define the deletion operation on circular words and show that it is strictly length-decreasing, hence never equivalent to a concatenation, that with empty contexts it realizes the inverse relation of the one-wheel concatenation, and that it can be simulated by Pixton circular splicing but not by the Head or the Păun one (Definition 8, Theorems 9 and 10).

These results concern single applications of the operations, thus the open problems on finite circular splicing *systems* mentioned above are not solved here and remain open, namely, the characterization of the circular languages, in particular the regular ones, generated by such systems, which is known only for restricted classes [4,6,7,18], and the comparison of the generative power of the systems based on the three definitions [6,19]; our Proposition 1 does not settle the latter, since the systems differ also in the structure and use of their sets of rules, however we hope that our results can be applied in further studies also in the direction to solve open problems about splicing systems.

The paper is organized as follows. Section 2 fixes the notations and recalls the necessary notions on words and circular words. Section 3 recalls the circular splicing operation in the definitions of Head, Păun and Pixton, compares these definitions with each other, and relates them to the one-wheel and to the two-wheel concatenations of circular words. Section 4 is devoted to the insertion and deletion operations: the circular insertion into circular words is defined and compared in Section 4.1 with the known circular insertion into linear words, with the Păun circular splicing and with the one-wheel concatenation, while Section 4.2 deals with the circular deletion. Section 5 discusses the implications of our results for systems generating circular languages. Finally, Section 6 summarizes the results and indicates directions for further research.

## 2. Preliminaries

The topic of the paper belongs to DNA computing, bioinformatics and models of DNA-based computing and information systems. In this section, we briefly recall some necessary preliminaries, and we also fix our notations. For any unexplained concepts, the reader is referred to [11,20,21].

Let Σ denote the alphabet, a finite non-empty set of symbols (or letters). A string (also called a word) is a finite sequence of letters. In this paper, the term `linear word’ is also used to contrast these words with the main subject of this paper, namely the circular or cyclic words, which are defined below. Further, as usual, Σ^*^ denotes the set of all words over the alphabet Σ. Let ℕ and ℕ_0_ denote the set of positive and non-negative integers, respectively. The length of a string *x* ∈ Σ^*^ is denoted by |*x*| and it is the length of the sequence, i.e., the number of symbols in *x*. Let *λ* denote the empty word; it is the only word having zero length. The *j^th^* letter of *x* ∈ Σ^*^ is denoted by *x_j_*, for *j* ∈ {1, …, |*x*|}. A word *w* ∈ Σ^*^ is a factor of *x* ∈ Σ^*^, written as *w* ∈ *Fact*(*x*), if there are two words *u*, *v* ∈ Σ^*^ such that *x* = *uwv*. Moreover, if *u* = *λ* (resp. *v* = *λ*), then *w* is also a prefix (resp. suffix) of *x*. For any non-negative integer and word *x*, the power of *x* is defined as follows: *x*^0^ = *λ*, further *x^k^*^+1^ = *xx^k^* for *k* ≥ 0. Two words *y* and *x* are called conjugates if there are words *v*,*u* ∈ Σ^*^ such that *y* = *vu* and *x* = *uv*.

A circular word *x*_○_ can be viewed graphically as the result of joining the first symbol of a linear word *x* to its last, thereby forming a closed loop. Circular words are also referred to as cyclic words and necklaces in the scientific literature. Unlike a linear word, a circular word has neither a distinguished beginning nor an end. Formally, a circular word associated with *x* is defined as the set of all conjugates (or, equivalently, all cyclic shifts) of *x*. This set-based representation is commonly referred to as the linearization of the circular word *x*_○_.

*x*_○_ = { *y* | *y* is a conjugate of *x*} (1)

For a circular word $x_{○}={\left(x_{1}\ldots x_{n}\right)}_{○}$, we define *Fact*(*x*_○_) to be the set that contains all substrings occurring in any conjugate of *x* (including the empty word). A circular word of length zero is called the empty circular word, and we may also write it as *λ*_○_. Further, let ${\left|x_{○}\right|}_{a}$ be the number of occurrences of *a* in *x*_○_, where *a* ∈ Σ, and it is defined to be the same as the number of occurrences of *a* in *x* (or in any other element of the set *x*_○_), i.e., ${\left|x_{○}\right|}_{a}={\left|x\right|}_{a}$. Although a circular word is formally represented as a set, all of its constituent words have the same length; hence, the notation |*x*_○_| is well-defined for their length. Conversely, the set *x*_○_ itself has a cardinality denoted by ‖*x*_○_‖, which does not exceed |*x*_○_|.

In the next sections, we also formally recall the splicing and insertion–deletion operations known in the literature for circular words.

## 3. Splicing Operation and Circular Splicing

Since circular splicing systems depend on the splicing rules used, various definitions are possible for them; however, some of them look very unnatural. As previously stated in [4,6,18], Head, Păun, and Pixton have provided three different definitions of the circular splicing operation. Generally, the main difference between the definitions of circular splicing lies in the way the splicing rules work.

**Definition 1** **(Head’s Definition).** *Let* Σ *be a finite alphabet, and* (*u*_1_, *x*, *u*_2_), (*u*_3_, *x*, *u*_4_) *be two triples of words in* Σ^*^ *sharing the same middle word x. Given two circular words* ${\left(z_{1}\right)}_{○}={\left(hu_{1}xu_{2}\right)}_{○}$ *and* ${\left(z_{2}\right)}_{○}={\left(ku_{3}xu_{4}\right)}_{○}$*, Head’s circular splicing operation produces* ${\left(hu_{1}xu_{4}ku_{3}xu_{2}\right)}_{○}$.

**Figure 1:**
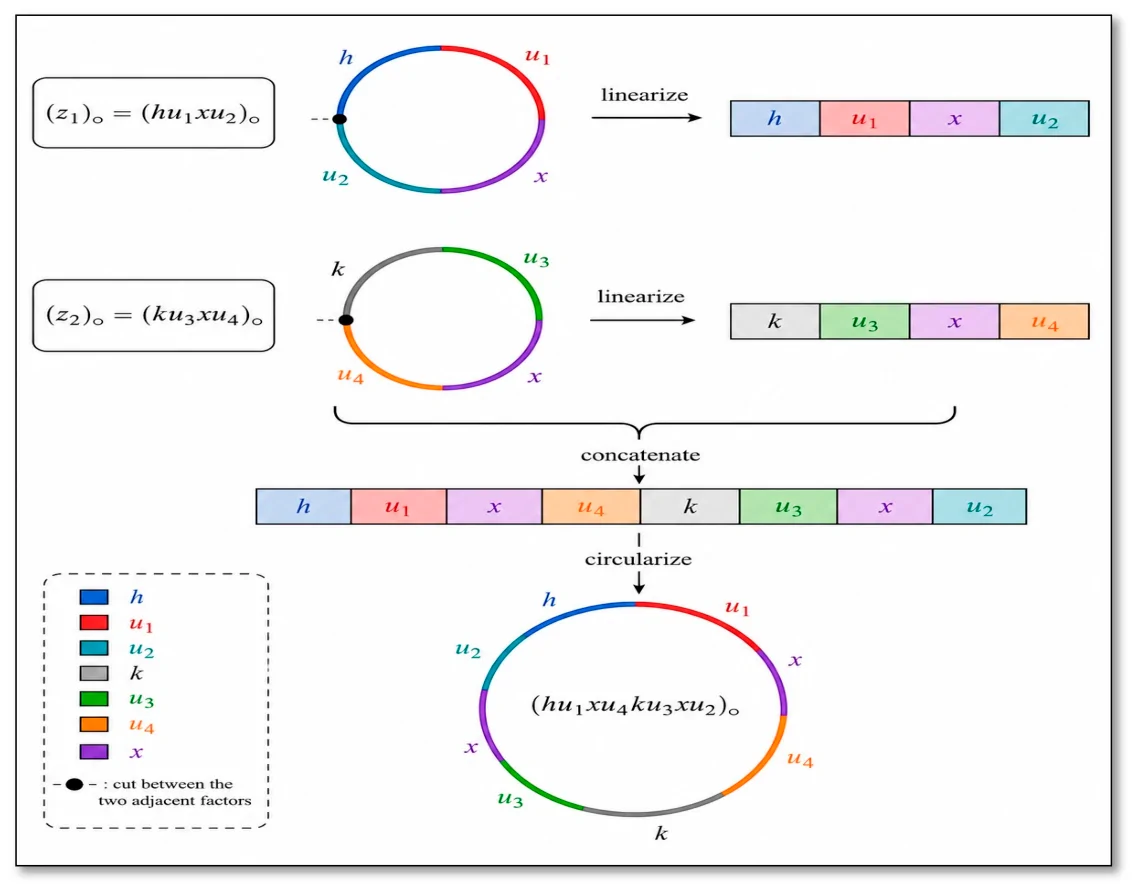
The Head Circular Splicing of Two Circular Words.

Although a splicing operation is applied prior to concatenation, the resulting word is identical to that obtained by concatenating alternative linearizations of the original circular words, namely *u*_2_*hu*_1_*x* and *u*_4_*ku*_3_*x*, respectively (see Figure 1).

As we shall see, applying a splicing operation to two circular words produces a circular word. However, the operation need not be applicable to every pair of input circular words; in such cases, no output circular word can be generated (obtained). Moreover, it may also happen that the input circular words have many occurrences of the subwords specified by the rule, and therefore the rule can be applied in more than one way, and thus it may be able to produce more than one possible result.

In our examples below, generally, the computations follow the same three steps prescribed by the corresponding definition (and also shown in the corresponding figure).

*(i)*  *Cut*, i.e., determine the linearizations of the two input circular words that have the form required by the rule;
*(ii)*  *Concatenate* the obtained linear words;
*(iii)*  *Circularize* the result.

Every possible choice in step (*i*) gives a result. Different choices may give the same circular word in step (*iii*). We display these steps in detail for Examples 1, 3 and 5; the other examples are computed analogously.

**Example** **1.** *Given the two triples of words (a, λ, b) and (b, λ, a), Head’s splicing operation applied to* ${\left(a^{2}b^{2}\right)}_{○}$ *and* (*ab*)_○_ *generates the circular word* ${\left(a^{3}b^{3}\right)}_{○}$*, and two copies of* (*ab*)_○_ *produce* ${\left(a^{2}b^{2}\right)}_{○}$*. However, using the same triple of words, Head’s splicing operation is not applicable to* ${\left(a^{2}b^{2}\right)}_{○}$ *and* (*aa*)_○_ *as the factor b is missing; hence, it generates the empty set. In addition, Head’s splicing operation applied to* (*abaab*)_○_ *and* (*aba*)_○_ *produces two different circular words* (*aaabbaab*)_○_ *and* (*abaaaabb*)_○_.

*For instance, in the first case of Example 1, the triples (a, λ, b) and (b, λ, a) require linearizations of the form hu*_1_*xu*_2_ = *hab and ku*_3_*xu*_4_ = *kba. The only conjugate of aabb ending with ab is baab (so h* = *ba), and the only conjugate of ab ending with ba is ba (so k* = *λ). By Definition 1, the result is* ${\left(hu_{1}xu_{4}ku_{3}xu_{2}\right)}_{○}={\left(\mathrm{ba}\cdot a\cdot a\cdot b\cdot b\right)}_{○}={\left(\mathrm{baaabb}\right)}_{○}={\left(a^{3}b^{3}\right)}_{○}$.

**Definition 2** **(Păun’s Definition).** *Let* Σ *be a finite alphabet, and let r* = (*u*_1_, *u*_2_; *u*_3_, *u*_4_)∈Σ^*^ × Σ^*^ × Σ^*^ × Σ^*^ *be a splicing rule. Given two circular words* ${\left(z_{1}\right)}_{○}={\left(u_{2}hu_{1}\right)}_{○}$ *and* ${\left(z_{2}\right)}_{○}={\left(u_{4}ku_{3}\right)}_{○}$*, the Păun circular splicing operation defined by r cuts the two circular words at the specified contexts, linearizes them to the words u*_2_*hu*_1_ *and u*_4_*ku*_3_*, concatenates the obtained linear words, and circularizes the result, producing the circular word* ${\left(u_{2}hu_{1}u_{4}ku_{3}\right)}_{○}$*. We denote this operation by* $({\left(z_{1}\right)}_{○},{\left(z_{2}\right)}_{○}){⊢}_{r}{\left(u_{2}hu_{1}u_{4}ku_{3}\right)}_{○}$.

**Example** **2.** *The Păun circular splicing operation is defined by the splicing rule r* = (*a*,*b*;*b*,*a*)*, and applied to* ${\left(a^{2}b^{2}\right)}_{○}$ *and* (*ab*)_○_ *generates the circular word* ${\left(a^{3}b^{3}\right)}_{○}$*. It produces* ${\left(a^{2}b^{2}\right)}_{○}$ *when applied to two copies of* (*ab*)_○_.

**Example** **3.** *Given the two triples of words* (*u*_1_*,x,u*_2_) = (*aa,ab,aa*) *and* (*u*_3_*,x,u*_4_) = (*bb,ab,bb*)*, Head’s splicing operation applied to* (*aaaaab*)_○_ *and* (*bbbbba*)_○_ *generates the circular word* (*aaabbbbbabaa*_○_*. However, using the same components for Păun circular splicing rule r* = (*u*_1_, *u*_2_; *u*_3_, *u*_4_) = (*aa,aa;bb,bb*)*, the Păun splicing operation applied to the same circular words produces four different circular words* (*aabaaabbabbb*)_○_, (*aabaaabbbabb*)_○_, (*aaabaabbabbb*)_○_ *and* (*aaabaabbbabb*)_○_.

*The computation is as follows. For Head’s operation, the triples (aa,ab,aa) and (bb,ab,bb) require linearizations of the form h⋅ aa⋅ ab⋅ aa and k⋅ bb⋅ ab⋅ bb. Since these words already have length 6, we get h = k = λ, and the unique suitable conjugates are aaabaa*∈ (*aaaaab*)_○_ *and bbabbb*∈ (*bbbbba*)_○_*. By Definition 1, the result is*

$${\left(hu_{1}xu_{4}ku_{3}xu_{2}\right)}_{○}={\left(\mathrm{aa}\cdot \mathrm{ab}\cdot \mathrm{bb}\cdot \mathrm{bb}\cdot \mathrm{ab}\cdot \mathrm{aa}\right)}_{○}={\left(\mathrm{aaabbbbbabaa}\right)}_{○}.$$

**Figure 2:**
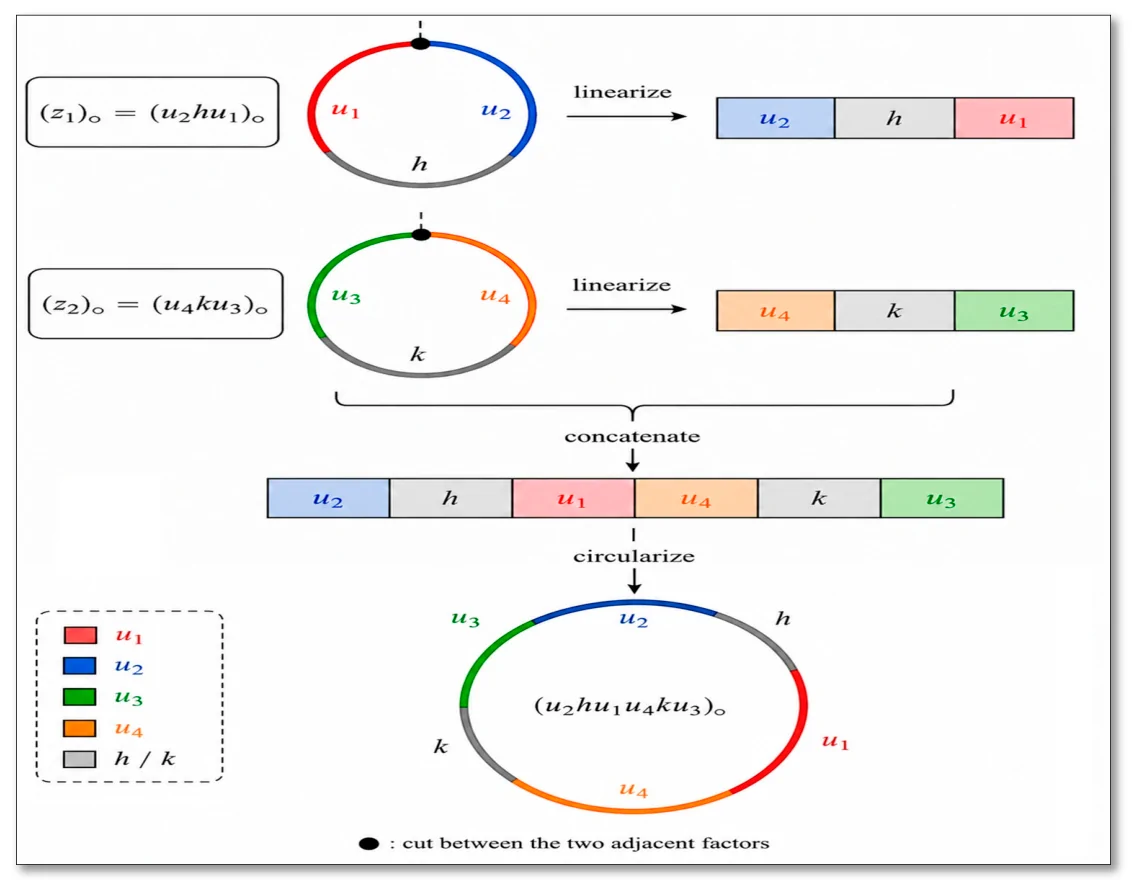
The Păun Circular Splicing of Two Circular Words.

*For the Păun rule r* = (*aa*, *aa*; *bb*, *bb*)*, by Definition* 2 *the linearizations must have the form u*_2_*hu*_1_ = *aa* ⋅ *h* ⋅ *aa and u*_4_*ku*_3_ = *bb* ⋅ *k* ⋅ *bb (refer to* Figure 2*)*. *Among the six conjugates of aaaaab*, *exactly two begin and end with aa*, *namely aaabaa* (*h* = *ab*) *and aabaaa* (*h* = *ba*). *Similarly, there are two options for linearizing the 6-letter long circular word* (*bbbbba*)_○_ *with prefix bb and suffix bb,* i.e.*, bbabbb* (*k* = *ab*) *and bbbabb* (*k* = *ba*)*. Combining them in the four possible ways gives the four distinct results:*
**Linearization of *x***_○_**Linearization of *y***_○_**Concatenation****Resulting Circular Word***aaabaa**bbabbb**aaabaabbabbb*(*aaabaabbabbb*)_○_*aaabaa**bbbabb**aaabaabbbabb*(*aaabaabbbabb*)_○_*aabaaa**bbabbb**aabaaabbabbb*(*aabaaabbabbb*)_○_*aabaaa**bbbabb**aabaaabbbabb*(*aabaaabbbabb*)_○_

*These four circular words are pairwise different, since none of the four linear words in the third column is a conjugate of another one. Notice that the difference in the results comes from the fact that in Head’s operation an additional parameter x = ab was used that has not appeared in the applied Păun splicing operation.*

From Examples 1 and 2, we observe that the definitions of Head and Păun yield the same resulting circular word when their respective splicing operations are applied to the circular words of these particular examples, but not for Example 3. However, the Head triples and the Păun rule in Example 3 do not correspond to each other as rules; Proposition 1 below shows that, under the appropriate translation of rules, the two operations yield the same results. Observe that although the splicing on linear words is applied in Head’s definition, it is applied in both ways, i.e., the splicing with its symmetric pair, and the concatenation of these two resulting strings yields a circular word that has as a subword a conjugate of both initial circular words, namely, *xu*_2_*hu*_1_ and *xu*_4_*ku*_3_, where the subword occurrence of *x* with the other subsequences written in the rules specify the positions where the cycles are cut before concatenating the result. By the Păun-type circular splicing, the rules also specify only how to cut the original circular words into linear words to obtain the new circular word after concatenating these two linear words, and thus, in the resulting circular word, a conjugate of each of the original words must appear. In Păun’s definition, this concatenation is more direct. In [6,19], these operations and computing systems based on them are compared. Now we complement the results shown there as follows:

**Proposition** **1.** *Let Σ be a finite alphabet, and let* (*u*_1_*,x,u*_2_) *and (u*_3_*,x, u*_4_) *be two triples of words in* Σ^*^ *sharing the same middle word x. Then, for any two circular words* ${\left(z_{1}\right)}_{○}$ *and* ${\left(z_{2}\right)}_{○}$*, Head’s circular splicing operation with these triples and the Păun circular splicing operation with the rule r* = (*u*_1_*x*, *u*_2_; *u*_3_*x*, *u*_4_) *produce the same set of circular words. In particular, if x* = *λ, then every Păun rule* (*u*_1_, *u*_2_; *u*_3_, *u*_4_) *is reproduced by the Head triples* (*u*_1_, *λ*, *u*_2_) *and* (*u*_3_, *λ*, *u*_4_) *and vice versa.*

**Proof.**  Applying the Head splicing operation with triples (*u*_1_*,x,u*_2_) and (*u*_3_*,x, u*_4_) to the circular words ${\left(z_{1}\right)}_{○}={\left(hu_{1}xu_{2}\right)}_{○}$ and ${\left(z_{2}\right)}_{○}={\left(ku_{3}xu_{4}\right)}_{○}$, by Definition 1, the resulting circular word is ${\left(hu_{1}xu_{4}ku_{3}xu_{2}\right)}_{○}$.

On the other hand, the Păun splicing rule *r* = (*u*_1_*x*,*u*_2_;*u*_3_*x*,*u*_4_) applying it to ${\left(z_{1}\right)}_{○}$ and ${\left(z_{2}\right)}_{○}$, by Definition 2, produces ${\left(u_{2}hu_{1}xu_{4}ku_{3}x\right)}_{○}$. Since *hu*_1_*xu*_4_*ku*_3_*xu*_2_ is a conjugate of *u*_2_*hu*_1_*xu*_4_*ku*_3_*x*, then ${\left(hu_{1}xu_{4}ku_{3}xu_{2}\right)}_{○}={\left(u_{2}hu_{1}xu_{4}ku_{3}x\right)}_{○}$.

Setting *x* = *λ* gives the second statement. □

We just proved in Proposition 1 that any circular word producible by a Head splicing can also be produced by a corresponding Păun splicing and vice versa.

For instance, based on the previous result, we complement Example 3.

**Example** **4.** *The Păun rule corresponding to the Head triples of Example 3 is* (*aaab*,*aa*;*bbab*,*bb*)*, which produces from* (*aaaaab*)_○_ *and* (*bbbbba*)_○_ *exactly the circular word* (*aaabbbbbabaa*)_○_*; conversely, the Păun rule* (*aa*,*aa*;*bb*,*bb*) *of Example 3 corresponds to the Head triples* (*aa*,*λ*,*aa*) *and* (*bb*,*λ*,*bb*)*, which yield the same four circular words we have described there.*

Differences between the two approaches arise only at the level of circular splicing systems (e.g., the structure and symmetry of the sets of rules, or the self-splicing included in Păun’s definition); see [6,19].

**Example** **5.** *Consider a Păun rule with all contexts empty, r* = (*λ*,*λ*;*λ*,*λ*)*. Splicing the two circular single words* (*a*)_○_ *and* (*b*)_○_ *results in* (*ab*)_○_*. Also, the Head triple pair* (*a*,*x*,*λ*) *and* (*b*,*x*,*λ*) *produces this from* (*a*)_○_ *and* (*b*)_○_*, with x* = *λ.*

It is important to remember that the splicing rules can be applied in two different ways according to Păun’s initial definition of the circular splicing operation as in [22] (one method has already been explained here, and the other, known as self-splicing, will not be discussed in this paper). Note that circular splicing is defined as length-increasing in both Head’s and Păun’s definitions; this means that when two circular words are spliced from one another, the spliced word’s length is equal to the sum of the lengths of the two words.

Pixton’s definition does not necessarily satisfy the same fact, as he defined the circular splicing system differently, allowing some rewriting, as follows:

**Definition 3** **(Pixton’s Definition).** *Let* Σ *be a finite alphabet, and let r* = (*α*,*α*′;*β*)∈Σ^*^ × Σ^*^ × Σ^*^ *be a splicing rule*. *Assume there exists a partner rule* $\bar{r}=(\alpha^{\prime },\alpha ;\beta \prime ){\in \Sigma }^{*}\times \Sigma^{*}\times \Sigma^{*}$ *associated with r. Given two circular words* ${\left(z_{1}\right)}_{○}={\left(\alpha \varepsilon \right)}_{○}$ *and* ${\left(z_{2}\right)}_{○}={\left(\alpha \prime \varepsilon \prime \right)}_{○}$*, the Pixton circular splicing operation defined by the rule pair* $\left(r,\;\bar{r}\right)$ *cuts the two circular words at the specified contexts, linearizes them to the words εα and ε′α′, substitutes the suffixes α,α′ by β, β′, concatenates the resulting linear words, and circularizes the result, producing the circular word* (*εβε*′*β*′)_○_*. We denote this operation by* $({\left(z_{1}\right)}_{○},{\left(z_{2}\right)}_{○}){⊢}_{\left(r,\bar{r}\right)}{\left(\varepsilon \beta \varepsilon \prime \beta \prime \right)}_{○}$.

**Figure 3:**
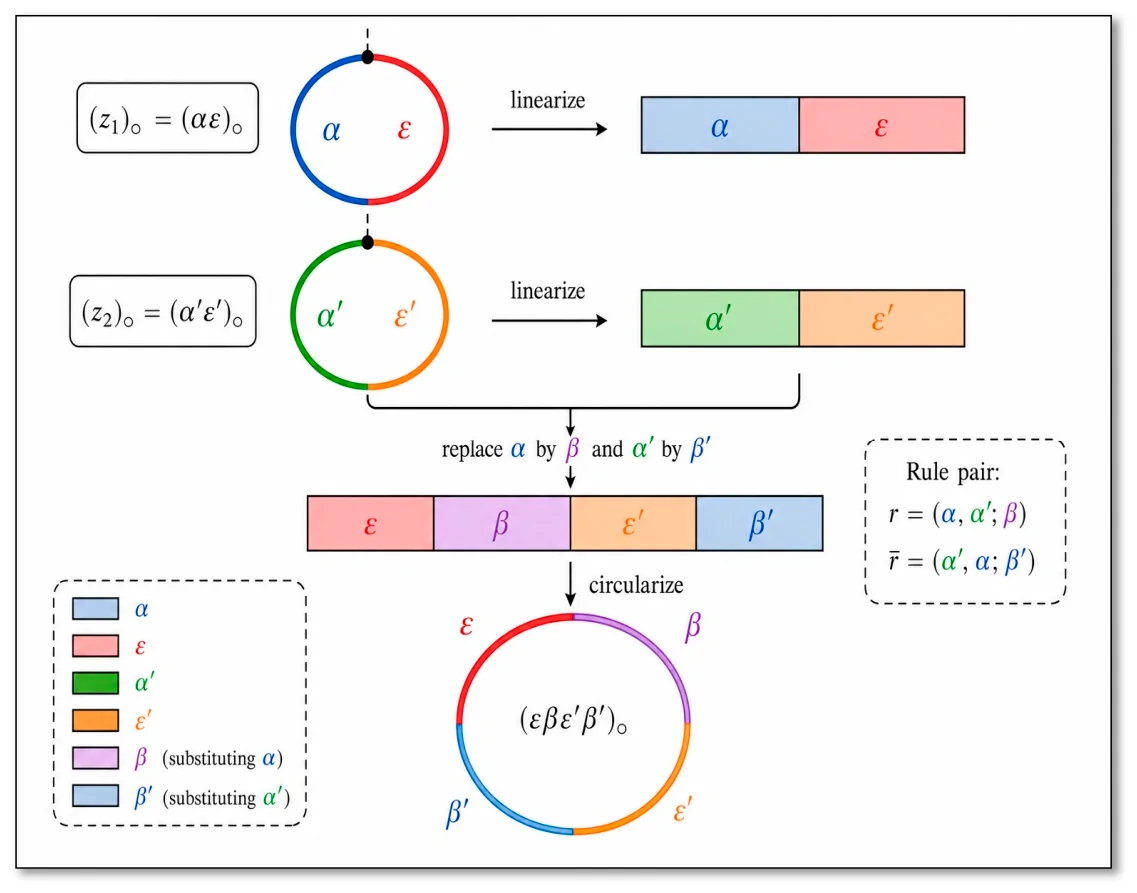
The Pixton Circular Splicing of Two Circular Words.

As Pixton’s definition allows some rewriting, step (*ii*) in the computation in our examples is modified accordingly: instead of a simple concatenation of the linearized words, one needs to replace the marked suffixes by the ones given in the rule (see Figure 3). We show an example below:

**Example** **6.** *The Pixton circular splicing operation defined by the splicing rules r* = (*a*^2^,*a*^2^*b*;*a*^2^) *and* $\bar{r}=(a^{2}b,a^{2};\lambda )$*, and applied to two copies of* ${\left(a^{4}b\right)}_{○}$ *, generates the circular word* ${\left(a^{6}b\right)}_{○}$*. In detail, the rule r requires a linearization of the first copy of* ${\left(a^{4}b\right)}_{○}$ *of the form αε with α* = *a*^2^*, giving ε* = *a*^2^*b, and its partner* $\bar{r}$ *requires a linearization of the second copy of the form α′ε′ with α′* = *a*^2^*. By Definition* 3*, the two circular words are linearized to εα* = *a*^2^*ba*^2^ *and ε′α′* = *a*^2^*a*^2^*b, the suffixes α and α′ are replaced by β* = *a*^2^ *and β′* = *λ, respectively, and the resulting words εβ* = *a*^2^*ba*^2^ *and ε′β′* = *a*^2^ *are concatenated and circularized, producing* ${\left(a^{2}ba^{4}\right)}_{○}={\left(a^{6}b\right)}_{○}$.

Pixton’s rules are symmetric by construction (every *r* has a partner $\bar{r}$), so in statements one should always mention the existence of the partner and that the pair is applied together. Moreover, for a rule *r* = (*α*,*α′*;*β*), there may exist distinct rules ${\bar{r}}_{i}=(\alpha^{\prime },\alpha ;\beta_{i}^{\prime })$ such that the pair $\left(r,\;{\bar{r}}_{i}\right)$ can be applied. Nevertheless, for the sake of simplicity and since no ambiguity arises from the context, we will adopt the notation $\left(r,\;\bar{r}\right)$ when a pair is applied.

**Example** **7.** *Let r* = (*ab*,*λ*;*a*^2^) *be a Pixton splicing rule over the alphabet* Σ = {*a*,*b*}, *having three partners* ${\bar{r}}_{1}=(\lambda ,\mathrm{ab};a)$, ${\bar{r}}_{2}=(\lambda ,\mathrm{ab};b)$ *and* ${\bar{r}}_{3}=(\lambda ,\mathrm{ab};\mathrm{ba})$. *Then, the Pixton circular splicing operation defined by the pair rules* $\left(r,\;{\bar{r}}_{1}\right)$, $\left(r,\;{\bar{r}}_{2}\right)$, $\left(r,\;{\bar{r}}_{3}\right)$ *and applied to* ${\left(a^{2}b\right)}_{○}$ *and* (*ab*)_○_ *produce respectively* ${\left(a^{5}b\right)}_{○}$ , ${\left(a^{4}b^{2}\right)}_{○}$ and ${\left(a^{5}b^{2}\right)}_{○}$.

In the next part of the section, we analyze the operations described above.

**Theorem** **1.** *Let* Σ *be an arbitrary finite alphabet, and let* $x_{○}={\left(x_{1}\ldots x_{n}\right)}_{○}$ *and* $y_{○}={\left(y_{1}\ldots y_{m}\right)}_{○}$ *be two circular words, with lengths at least 2 and with indices taken modulo n and m respectively (so x_n_*_+1_ = *x*_1_ *and y_m_*_+1_ = *y*_1_). *For fixed indices i* ∈ {1,…,*n*} *and j* ∈ {1,…,*m*}, *the following nine Păun splicing rules applied to x*_○_ *and y*_○_ *generate the same circular word if* ${\left|x_{○}\right|}_{x_{i}}={\left|x_{○}\right|}_{x_{i+1}}={\left|y_{○}\right|}_{y_{j}}={\left|y_{○}\right|}_{y_{j+1}}=1$:
*r*_1_ = (*x_i_*,*x_i_*_+1_;*y_j_*,*y_j_*_+1_),*r*_2_ = (*x_i_*,*λ*;*y_j_*,*y_j_*_+1_),*r*_3_ = (*λ*,*x_i_*_+1_;*y_j_*,*y_j_*_+1_),*r*_4_ = (*x_i_*,*x_i_*_+1_;*y_j_*,*λ*),*r*_5_ = (*x_i_*,*x_i_*_+1_;*λ*,*y_j_*_+1_),*r*_6_ = (*x_i_*,*λ*;*y_j_*,*λ*),*r*_7_ = (*x_i_*,*λ*;*λ*,*y_j_*_+1_).*r*_8_ = (*λ*,*x_i_*_+1_;*y_j_*,*λ*),*r*_9_ = (*λ*,*x_i_*_+1_;*λ*,*y_j_*_+1_).

**Proof.**  Since *n*,*m* > 1, the indices *i* and *i* + 1 and *j* and *j* + 1, respectively, denote neighbor positions in the two words. Assume ${\left|x_{○}\right|}_{x_{i}}={\left|x_{○}\right|}_{x_{i+1}}={\left|y_{○}\right|}_{y_{j}}={\left|y_{○}\right|}_{y_{j+1}}=1$, meaning that the letters *x_i_* and *x_i_*_+1_ (resp. *y_j_* and *y_j_*_+1_) occur only once in *x*_○_ (resp. *y*_○_). Thus, the conjugate of *x* that starts at *x_i_*_+1_ (or ends at *x_i_*) is the unique word *x_i_*_+1_ … *x_n_x*_1_ … *x_i_*, and likewise, the conjugate of *y* that starts at *y_j_*_+1_ (or ends at *y_j_*) is also uniquely defined *y_j_*_+1_ … *y_m_y*_1_ … *y_j_*. Thus, each of the nine rules applied to *x*_○_ and *y*_○_ cuts the two circular words at the same positions, and therefore, generates the same circular word $z_{○}={\left(x_{i+1}\ldots x_{n}x_{1}\ldots x_{i}y_{j+1}\ldots y_{m}y_{1}\ldots y_{j}\right)}_{○}$. □

Note here that the hypothesis |*x*_○_|, |*y*_○_| ≥ 2 cannot be dropped. For example, if |*x*_○_| = 1, then indices taken modulo *n* give *x_i_* = *x_i_*_+1_, hence any rule whose first pair is (*x_i_*,*x_i_*_+1_) requires the factor *x_i_x_i_* in *x*_○_ and is therefore inapplicable, while rules with *λ* in that position remain applicable. For instance, with *x*_○_ = (*a*)_○_, *y*_○_ = (*bcd*)_○_ and *i* = *j* = 1, the rule *r*_1_ = (*x_i_*,*x_i_*_+1_;*y_j_*,*y_j_*_+1_) = (*a*,*a*;*b*,*c*) generates nothing, whereas *r*_2_ = (*x_i_*,*λ*;*y_j_*,*y_j_*_+1_) = (*a*,*λ*;*b*,*c*) produces (*acdb*)_○_. Therefore, the results obtained by *r*_1_, …, *r*_9_ do not coincide, and the same argument applies symmetrically when |*y*_○_| = 1. Further, the scenario is similar in the case when one or both of the circular words are the empty word. In general, the Păun splicing rules *r* = (*x_i_*,*x_i_*_+1_;*y_j_*,*y_j_*_+1_) and *r′* = (*λ*,*λ*;*y_j_*,*y_j_*_+1_) applied to *x*_○_ and *y*_○_ do not necessarily give the same results. On the one hand, while the rule *r′* can cut and linearize *x*_○_ to obtain any conjugate of *x*, the rule *r* cuts and linearizes *x*_○_ obtaining, e.g., *x_i_*_+1_ … *x_n_x*_1_ … *x_i_*. However, if *x*_○_ is built up only by one of the letters, i.e., it is of the form ${\left(a^{n}\right)}_{○}$ for a letter *a* ∈ Σ, then there is only one linear word representing it, and any cut will lead to this sole word.

**Example** **8.** *Consider the circular words* $x_{○}={{\left(x_{1}x_{2}x_{3}x_{4}\right)}_{○}=\left(\mathrm{abcd}\right)}_{○}$ *and* $y_{○}={\left(y_{1}y_{2}\right)}_{○}{=\left(12\right)}_{○}$ *in the alphabet* Σ = {*a*,*b*,*c*,*d*,1,2} ={*a*,*b*,*c*,*d*,1,2}*. For fixed indices i* = 3 *and j* = 2 *(*${x_{i}=x}_{3}=c$ *and* ${y_{j}=y}_{2}=2$*), each of the nine Păun splicing rules in Theorem* 1 *applied to x*_○_ *and y*_○_ *generates exactly the same circular word* ${\left(x_{4}x_{1}x_{2}x_{3}y_{1}y_{2}\right)}_{○}={\left(\mathrm{dabc}12\right)}_{○}$.

*On the other hand, consider the circular word* $z_{○}={{\left(z_{1}z_{2}z_{3}z_{4}z_{5}\right)}_{○}=\left(\mathrm{abcad}\right)}_{○}$ *where the letter a occurs twice. Fixing the indices i* = 1 *and j* = 2 *(*${z_{i}=z}_{1}=a$ *and* ${y_{j}=y}_{2}=2$*), the Păun circular splicing rule r*_1_ = (*a*,*b*;2,1) *applied to z*_○_ *and y*_○_ *generates the circular word* (*bcada*12)_○_*, while the splicing rule r*_2_ = (*a*,*λ*;2,1) *applied to z*_○_ *and y*_○_ *generates two different circular words* (*bcada*12)_○_ *and* (*dabca*12)_○_.

*In addition, the factor ab in the circular word* $w_{○}={{\left(w_{1}w_{2}w_{3}w_{4}w_{5}w_{6}\right)}_{○}=\left(\mathrm{abcabd}\right)}_{○}$ *occurs twice. However, the Păun circular splicing rule r*_1_ = (*a*,*b*;2,1) *applied to w*_○_ *and y*_○_ *generates two different circular words* (*bcabda*12)_○_ *and* (*bdabca*12)_○_.

We recall the definitions of one-wheel and two-wheel concatenations of circular words from [17] to allow comparing them to the splicing operations.

**Definition** **4.** *Let x*_○_ *and y*_○_ *be two circular words; their two-wheel concatenation is the sequence of the circular words obtained by:*

*x*_○_*y*_○_ = *x*_○_∙*y*_○_ = { *z* | *z* = *uv* where *u* ∈ *x*_○_ and *v* ∈ *y*_○_} (2)

**Definition** **5.** *Let x*_○_ *and y*_○_ *be two circular words; their one-wheel concatenation is:*

*x*_○_⊙*y*_○_ = {*w* ∈ *z*_○_ | *z* = *uv* where *u* ∈ *x*_○_ and *v* ∈ *y*_○_ } (3)

*It produces all cyclic permutations of the concatenation of any conjugate of x and any conjugate of y*.

In [17], it was proved that the one-wheel concatenation is an operation that is commutative but not associative, while the two-wheel concatenation is associative but not commutative.

**Theorem** **2.** *Let* Σ *be an arbitrary finite alphabet, and let* $x_{○}={\left(x_{1}\ldots x_{n}\right)}_{○}$ *and* $y_{○}={\left(y_{1}\ldots y_{m}\right)}_{○}$ *be any arbitrary circular words over* Σ *with indices taken modulo n and m respectively (so x_n_*_+1_ = *x*_1_ *and y_m_*_+1_ = *y*_1_*). Then, considering any Păun circular splicing operation r* = (*u*_1_, *u*_2_; *u*_3_, *u*_4_) *the set of circular words obtained is a subset of the set obtained by the one-wheel concatenation applied to x*_○_ *and y*_○_*. Furthermore, applying all possible Păun circular splicing operations that produce a non-empty set, the union of the obtained circular words coincides with the set obtained by the one-wheel concatenation.*

**Proof.**  We prove the two inclusions separately. Since *x*_○_ is the set of all cyclic permutations of *x* = *x*_1_ … *x_n_*, we have:

*x*_○_ = {*x_i_*_+1_ … *x_n_x*_1_ … *x_i_* | *i* = 1,…,*n*}.

Similarly,

*y*_○_ = {*y_j_*_+1_ … *y_m_y*_1_ … *y_j_* | *j* = 1,…,*m*}.

And by Definition 5, *x*_○_⊙*y*_○_ is the set of circular words ${\left(x_{i+1}\ldots x_{n}x_{1}\ldots x_{i}y_{j+1}\ldots y_{m}y_{1}\ldots y_{j}\right)}_{○}$ for all *i* = 1,…,*n* and *j* = 1,…,*m*. The proof continues with the verification of the following two claims:

**Claim** **1.** *For every Păun rule r* = (*u*_1_, *u*_2_; *u*_3_, *u*_4_)*, the set of circular words produced by r from x*_○_ *and y*_○_ *is contained in x*_○_⊙*y*_○_.

**Proof** **of the Claim.** Indeed, by Definition 2, every application of *r* to *x*_○_ and *y*_○_ is determined by a pair (*h*,*k*) ∈ Σ^*^ × Σ^*^ such that $x_{○}={\left(u_{2}hu_{1}\right)}_{○}$ and $y_{○}={\left(u_{4}ku_{3}\right)}_{○}$, and it produces ${\left(u_{2}hu_{1}u_{4}ku_{3}\right)}_{○}$. Several such pairs may exist, one for each occurrence of the specified subwords, and the rule is applied once for each of them; however, for every one of them the word *u*_2_*hu*_1_ is a conjugate of *x* and *u*_4_*ku*_3_ is a conjugate of *y*, so that the produced circular word is the circularization of the concatenation of a conjugate of *x* and a conjugate of *y*, i.e., an element of *x*_○_⊙*y*_○_. If no such pair exists, the rule produces the empty set, which is a subset of *x*_○_⊙*y*_○_ as well. This proves the claim, the first part of the statement of the theorem. □

**Claim** **2.** *Every element of x*_○_⊙*y*_○_ *is produced by some Păun circular splicing rules.*

**Proof** **of the Claim.** Let ${z_{○}=\left(x_{i+1}\ldots x_{n}x_{1}\ldots x_{i}y_{j+1}\ldots y_{m}y_{1}\ldots y_{j}\right)}_{○}$ be an arbitrary element of *x*_○_⊙*y*_○_, with *i* and *j* fixed. Assume first *n*,*m* ≥ 2, and consider the Păun circular splicing rule *r* = (*x_i_*,*x_i_*_+1_;*y_j_*,*y_j_*_+1_). It is applicable to *x*_○_ and *y*_○_, since $x_{○}={\left(x_{i+1}hx_{i}\right)}_{○}$ with *h* = *x_i_*_+2_ … *x_n_x*_1_ … *x_i_*_−1_ and, analogously, $y_{○}={\left(y_{j+1}ky_{j}\right)}_{○}$ with *k* = *y_j_*_+2_ … *y_m_y*_1_ … *y_j_*_−1_. The corresponding application produces exactly *z*_○_. If the factor *x_i_x_i_*_+1_ occurs at other positions of *x*_○_, and/or *y_j_y_j_*_+1_ at other positions of *y*_○_, then *r* produces further circular words, which, by Claim 1, also belong to *x*_○_⊙*y*_○_; this does not affect the argument, as we only need to show that *z*_○_ to be produced.

If *n* ≤ 1, then *x*_○_ has a unique linearization, and we replace the first pair of the rule by (*λ*,*λ*); analogously, if *m* ≤ 1, we replace the second pair by (*λ*,*λ*). In each case, the obtained rule is applicable and produces *z*_○_. □

Now, continuing the proof of the theorem, by Claim 1, the union of the sets produced by all the Păun rules is contained in *x*_○_⊙*y*_○_, and by Claim 2 it contains it in the other way around; hence the two sets are equal, which proves the second part of the statement of the theorem. □

Theorem 2 is a characterization rather than a technically involved result: once the splicing rules are read as prescriptions of the positions at which the input circular words may be cut, both inclusions follow from Definitions 2 and 5 as it is shown above. Its role in this paper is to fix the exact ceiling of one application of the operations: namely, that whatever the contexts are, a single step cannot produce more than the one-wheel concatenation of its inputs, and that the whole of it is obtained when no site is prescribed. All the subsequent results rest on this bound: Corollary 1 and Proposition 2 describe how the produced sets vary with the contexts, Theorems 7 and 9 characterize the cases in which the two-wheel concatenation is reached instead, Theorems 7 and 8 place the circular insertion in the same picture, and Corollary 2 transfers the bound to the generated circular languages.

**Example** **9.** *Consider the circular words* $x_{○}={{\left(x_{1}x_{2}x_{3}\right)}_{○}=\left(\mathrm{abc}\right)}_{○}$ *and* $y_{○}={\left(y_{1}y_{2}\right)}_{○}{=\left(12\right)}_{○}$*, with each of the following Păun circular splicing rules* (*b*,*c*;1,2)*,* (*b*,*λ*;1,2)*,* (*λ*,*c*;1,*λ*)*, over the alphabet* Σ = {*a*,*b*,*c*,1,2}*. Each rule applied to x*_○_ *and y*_○_ *corresponds to the same cut-and-paste site, and generates the same circular word* (*cab*21)_○_.

**Corollary** **1.** *The Păun circular splicing rule* (*λ*,*λ*;*λ*,*λ*) *applied to any arbitrary circular words x*_○_ *and y*_○_ *generates their one-wheel concatenation.*

**Proof.**  By Claim 1 in the proof of Theorem 2, the set of circular words produced by this rule is contained in *x*_○_⊙*y*_○_. Conversely, let *z*_○_ ∈ *x*_○_⊙*y*_○_, so that *z* is a conjugate of *uv* for some *u* ∈ *x*_○_ and *v* ∈ *y*_○_. Since *λ* is both a prefix and a suffix of every word, we have *x*_○_ = (*λuλ*)_○_ and *y*_○_ = (*λvλ*)_○_, i.e., the rule (*λ*,*λ*;*λ*,*λ*) is applicable with *h* = *u* and *k* = *v*, and by Definition 2 it produces (*uv*)_○_ = *z*_○_. Hence every element of *x*_○_⊙*y*_○_ is obtained. □

Corollary 1 concerns the rule with empty contexts, which is the least restrictive one, and one may ask whether this case is not too degenerate to be informative. Two observations show that it is not.

First, the inclusion proved in the first part of Theorem 2 holds for arbitrary rules: whatever the contexts are, the circular words produced by a Păun splicing are always among those given by the one-wheel concatenation, and the same holds for the circular insertion (Theorem 7), where the contexts are arbitrary as well. The empty contexts are needed only for the reverse inclusion, i.e., to reach every element of the concatenation. The equivalence results are therefore not about one particular rule, but the two ends of a general property: the one-wheel concatenation is an upper bound for all these operations, which is attained exactly when no site is prescribed.

Second, between these two ends the behaviour is monotone with respect to the contexts, in the following sense.

**Proposition** **2.** *Let r* = (*u*_1_, *u*_2_; *u*_3_, *u*_4_) *and* $r^{\prime }=\left(u_{1}^{\prime },u_{2}^{\prime };u_{3}^{\prime },u_{4}^{\prime }\right)$ *be two Păun splicing rules such that* $u_{1}^{\prime }$ *is a suffix of u*_1_*,* $u_{2}^{\prime }$ *is a prefix of u*_2_*,* $u_{3}^{\prime }$ *is a suffix of u*_3_ *and* $u_{4}^{\prime }$ *is a prefix of u*_4_*. Then, for any circular words x*_○_ *and y*_○_*, the set of circular words produced by r is a subset of the set produced by r′.*

**Proof.**  Assume that *r* is applicable to *x*_○_ and *y*_○_, i.e., $x_{○}={\left(u_{2}hu_{1}\right)}_{○}$ and $y_{○}={\left(u_{4}ku_{3}\right)}_{○}$ for some *h*,*k*∈Σ^*^, then a produced circular word being ${\left(u_{2}hu_{1}u_{4}ku_{3}\right)}_{○}$. By the hypothesis, there are words *p*,*p′*,*q*,*q′*∈Σ^*^ with $u_{1}=pu_{1}^{\prime }$, $u_{2}=u_{2}^{\prime }p^{\prime }$, $u_{3}=qu_{3}^{\prime }$ and $u_{4}=u_{4}^{\prime }q^{\prime }$. Putting *h′* = *p′hp* and *k′* = *q′kq*, we obtain $u_{2}hu_{1}=u_{2}^{\prime }h^{\prime }u_{1}^{\prime }$ and $u_{4}ku_{3}=u_{4}^{\prime }k^{\prime }u_{3}^{\prime }$. Hence $x_{○}={\left(u_{2}^{\prime }h^{\prime }u_{1}^{\prime }\right)}_{○}$ and $y_{○}={\left(u_{4}^{\prime }k^{\prime }u_{3}^{\prime }\right)}_{○}$, so *r′* is also applicable to them, and by Definition 2 it can produce ${\left(u_{2}^{\prime }h^{\prime }u_{1}^{\prime }u_{4}^{\prime }k^{\prime }u_{3}^{\prime }\right)}_{○}={\left(u_{2}hu_{1}u_{4}ku_{3}\right)}_{○}$, i.e., the same circular word. □

In particular, taking $u_{1}^{\prime }=u_{2}^{\prime }=u_{3}^{\prime }=u_{4}^{\prime }=\lambda$ gives the inclusion of Corollary 1, so the rule (*λ*, *λ*; *λ*, *λ*) is the maximum of this ordering, while prescribing longer sites yields smaller sets of products; the results of Theorem 1 are the special case in which the restriction imposed by the contexts does not change the set at all. Together with Claim 2 in the proof of Theorem 2, which recovers the whole one-wheel concatenation as the union over all rules, this describes how the produced sets vary with the contexts, and not only at the degenerate end. Moreover, the characterizations of the forthcoming Theorems 7 and 9 are not restricted to empty contexts either: there the input circular words are forced to be of a special form, whereas the contexts may be arbitrary factors of them.

Beyond the one-wheel concatenation, which combines conjugates of two circular words into a single circular structure, the two-wheel concatenation preserves the independent circularity of each component before concatenation. However, unlike circular splicing, the result of a two-wheel concatenation is not necessarily a circular word, since the operation may yield a non-circular configuration. As the cases where one-wheel and two-wheel concatenations coincide were investigated in [17], it is now natural to examine the conditions under which the outcomes of Păun splicing coincide with those of two-wheel concatenation. Such a correspondence provides a precise structural characterization of when the generative power of Păun’s operation matches the two-wheel concatenation of two circular words. The next technical lemma and its proof are helpful to establish our further results.

**Lemma** **1.** *Let x*_○_ *and y*_○_ *be two circular words over* Σ*. Then, the two-wheel concatenation of x*_○_ *and y*_○_ *yields a circular word language if and only if x*,*y* ∈ {*a*}^*^ *for some a* ∈ Σ*, or x*_○_ = *λ*_○_*, or y*_○_ = *λ*_○_.

**Proof.**  (Necessary condition) Assume that *x*_○_∙*y*_○_ is a circular word language (i.e., closed under conjugation), and suppose that *x* ≠ *λ* and *y* ≠ *λ*. Let *A* and *B* denote the sets of letters occurring in *x* and in *y*, respectively. We distinguish three cases, which cover all the possibilities.

- Case 1: there is a letter *a* that is in *A* but not in *B*. Every element of *x*_○_∙*y*_○_ is of the form *uv* with *u* ∈ *x*_○_ and *v* ∈ *y*_○_, with *v* ≠ *λ*. So, its last letter is a letter of *B*; hence, no element of the set ends with *a*. On the other hand, *a* occurs in *x*, so it occurs in every element of the set, and therefore some conjugate of such an element ends with *a*. By the assumed closure under conjugation, this conjugate belongs to the set, a contradiction.
- Case 2: there is a letter *b* that is in *B* but not in *A*. The argument is symmetric: every element *uv* of the set begins with a letter of *A* because *u* ≠ *λ*, so no element begins with *b*, while some conjugate of an element does; again, a contradiction.
- Case 3: *A* = *B* and ‖*A*‖ ≥ 2. Fix *a* ∈ *A*, and let *n* = *max*{*k* ∈ ℕ_0_ | *a^k^* ∈ *Fact*(*x*_○_)} and *m* = *max*{*k* ∈ ℕ_0_ | *a^k^* ∈ *Fact*(*y*_○_)}; both are well defined and *n*,*m* ≥ 1, since *a* occurs in *x* and in *y*, while *n* < |*x*| and *m* < |*y*|, since *x* and *y* contain a letter different from *a*. As the occurrences of *a^n^* in the circular word *x*_○_ are preceded and followed by letters different from *a*, there is a linearization *u* ∈ *x*_○_ ending with *a^n^*, and, analogously, a linearization *v* ∈ *y*_○_ beginning with *a^m^*. Then *uv* ∈ *x*_○_∙*y*_○_ has the factor *a^n^*^+*m*^, and therefore it has a conjugate *z* beginning with *a^n^*^+*m*^. By the assumed closure under conjugation, *z* ∈ *x*_○_∙*y*_○_, i.e., *z* = *u′v′* for some *u′* ∈ *x*_○_ and *v′* ∈ *y*_○_. Since *u′* is a conjugate of *x*, it is not a power of *a* and it is not empty, so the maximal prefix of *z* of the form a^*^ is the maximal prefix of *u′* of this form, whose length is at most *n*. This contradicts the fact that *z* begins with *a^n^*^+*m*^, because *m* ≥ 1.

Consequently, if *x* ≠ *λ* and *y* ≠ *λ*, then *A* = *B* and ‖*A*‖ = 1, i.e., *x*,*y* ∈ {*a*}^*^ for some *a* ∈ Σ; otherwise, *x*_○_ = *λ*_○_ or *y*_○_ = *λ*_○_.

(Sufficient condition) If *x*,*y* ∈ {*a*}^*^, then *x*_○_∙*y*_○_ = {*a*^|*x*|+|*y*|^}, and if one of the circular words is *λ*_○_, then *x*_○_∙*y*_○_ is *x*_○_ or *y*_○_; all these sets are circular word languages, hence the two-wheel concatenation is a circular word language. □

**Theorem** **3.** *Let* Σ *be an arbitrary finite alphabet, and let* $x_{○}={\left(x_{1}\ldots x_{n}\right)}_{○}$ *and* $y_{○}={\left(y_{1}\ldots y_{m}\right)}_{○}$ *be any two arbitrary circular words with indices taken modulo n and m respectively (so x_n_*_+1_ = *x*_1_ *and y_m_*_+1_ = *y*_1_*). Then, the Păun circular splicing applied to them has the same result as their two-wheel concatenation if and only if (at least) one of the following three cases holds:*

1. *x*,*y* ∈ {*a*}^*^ *for any a* ∈ Σ, *and the rule r* = (*u*_1_, *u*_2_; *u*_3_, *u*_4_) *has the properties*
  - *u*_1_*u*_2_ ∈ { *a^p^* | *p* ∈ ℕ_0_ *with* 0 ≤ *p* ≤ |*x*|} *and*
  - *u*_3_*u*_4_ ∈ { *a^q^* | *q* ∈ ℕ_0_ *with* 0 ≤ *q* ≤ |*y*|};
2. $x_{○}={\left(x_{1}\ldots x_{n}\right)}_{○}\neq \lambda_{○}$ *, y*_○_ = *λ*_○_ *and r* = (*u*_1_,*u*_2_;*λ*,*λ*) *where u*_1_*u*_2_ ∈ *Fact*(*x*_○_)*;*
3. *x*_○_ = *λ*_○_*,*  $y_{○}={\left(y_{1}\ldots y_{m}\right)}_{○}\neq \lambda_{○}$ *and r* = (*λ*, *λ*; *u*_3_, *u*_4_) *where u*_3_*u*_4_ ∈ *Fact*(*y*_○_).

**Proof.**  (Necessary condition) Assume that the Păun circular splicing operation applied to *x*_○_ and *y*_○_ results in *x*_○_∙*y*_○_. Since this operation produces a circular word language, *x*_○_∙*y*_○_ is one, and by Lemma 1, either *x*,*y* ∈ {*a*}^*^ for some *a* ∈ Σ, or one of the two circular words is *λ*_○_.

Let *x*,*y* ∈ {*a*}^*^. Then, *x*_○_∙*y*_○_ = {*a*^|*x*|+|*y*|^}, and this must be produced by the Păun splicing rule *r* = (*u*_1_, *u*_2_; *u*_3_, *u*_4_) so that $x_{○}={\left(u_{2}hu_{1}\right)}_{○}$ and $y_{○}={\left(u_{4}ku_{3}\right)}_{○}$ for some *h*,*k*∈Σ^*^. Hence:

*u*_1_*u*_2_ ∈ *Fact*(*x*_○_) = *Fact*(*a*^|*x*|^) and *u*_3_*u*_4_ ∈ *Fact*(*y*_○_) = *Fact*(*a*^|*y*|^).

Thus, we obtain: *u*_1_*u*_2_ ∈ { *a^p^* | *p* ∈ ℕ_0_ with 0 ≤ *p* ≤ |*x*|} and *u*_3_*u*_4_ ∈ { *a^q^* | *q* ∈ ℕ_0_ with 0 ≤ *q* ≤ |*y*|}. This yields case (1), including the case when both of *x*_○_ and *y*_○_ are the empty circular word.

On the other hand, if $x_{○}={\left(x_{1}\ldots x_{n}\right)}_{○}\neq \lambda_{○}$ and *y*_○_ = *λ*_○_, then *x*_○_∙*y*_○_ is just the set of linear words equal to conjugates of *x*. For equality with the result obtained by the Păun splicing rule, we must be able to produce the circular word *x*_○_. This can be done by any applicable Păun splicing rule, i.e., by any rule of the form (*u*_1_,*u*_2_;*λ*,*λ*), where *u*_1_*u*_2_ ∈ *Fact*(*x*_○_) (as a special case, as we have shown before, it can be done by the rule (*λ*,*λ*;*λ*,*λ*)). Thus, case (2) holds.

Analogously, having *x*_○_ = *λ*_○_ and $y_{○}={\left(y_{1}\ldots y_{m}\right)}_{○}\neq \lambda_{○}$ yields that the rules of the form (*λ*,*λ*;*u*_3_,*u*_4_) with *u*_3_*u*_4_ ∈ *Fact*(*y*_○_) satisfy case (3). This completes the necessity direction.

(Sufficient condition) We now show that each of the listed cases is sufficient to guarantee equality.

Case 1: Let *x*,*y* ∈ {*a*}^*^ for any *a* ∈ Σ, and assume that the splicing rule *r* = (*u*_1_, *u*_2_; *u*_3_, *u*_4_) has the property

*u*_1_*u*_2_ ∈ { *a^p^* | *p* ∈ ℕ_0_ with 0 ≤ *p* ≤ |*x*|} and *u*_3_*u*_4_ ∈ { *a^q^* | *q* ∈ ℕ_0_ with 0 ≤ *q* ≤ |*y*|}.

Take *h* = *a*^|*x*|−*p*^ and *k* = *a*^|*y*|−*q*^ so that *x* = *u*_2_*hu*_1_ and *y* = *u*_4_*ku*_3_. Then, applying *r* yields ${\left(u_{2}hu_{1}u_{4}ku_{3}\right)}_{○}=\left\{a^{\left|x\right|+\left|y\right|}\right\}=x_{○}\cdot y_{○}$. Hence, the result.

Case 2: Since *u*_1_*u*_2_ ∈ *Fact*(*x*_○_), there exists an element of *x*_○_ of the form $x_{○}={\left(u_{2}hu_{1}\right)}_{○}$ for some *h* ∈ Σ^*^. Moreover, *y*_○_ = *λ*_○_ = (*λλλ*)_○_. Applying the Păun splicing rule *r* = (*u*_1_,*u*_2_;*λ*,*λ*), the two circular words are linearized as *u*_2_*hu*_1_ and *λ*, respectively. By Definition 2, the resulting circular word is ${\left(u_{2}hu_{1}\right)}_{○}=x_{○}$. On the other hand, by the definition of the two-wheel concatenation, *x*_○_∙*y*_○_ = *x*_○_∙*λ*_○_ = *x*_○_. Hence, the Păun circular splicing applied to *x*_○_ and *y*_○_ has the same result as their two-wheel concatenation.

The proof of Case 3 is analogous to Case 2: in fact, *λ*_○_ is the unit element of the two-wheel concatenation; therefore, the result will be exactly *x*_○_∙*y*_○_, thus the equality. □

**Example** **10.** *The following cases illustrate Theorem* 3 *and show that its conditions cannot be weakened. Let* Σ = {*a*,*b*,*c*}.

- *Let* $x_{○}={\left(a^{2}\right)}_{○}$, $y_{○}={\left(a^{3}\right)}_{○}$ *and r* = (*a*,*a*;*λ*,*a*). *Here u*_1_*u*_2_ = *a*^2^ *and u*_3_*u*_4_ = *a, so Case* 1 *holds, and r produces* ${\left(a^{5}\right)}_{○}=\left\{a^{5}\right\}=x_{○}\cdot y_{○}$. *However, for r*′ = (*a*^3^,*a*;*λ*,*a*) *we have u*_1_*u*_2_ = *a*^4^, *whose length exceeds* |*x*| = 2; *hence a*^4^ ∉ *Fact*(*x*_○_), *the rule is not applicable, and it produces the empty set instead of x*_○_∙*y*_○_. *Thus, the length bound in Case* 1 *is necessary.*
- *Let x*_○_ = (*abc*)_○_, *y*_○_ = *λ*_○_ *and r* = (*b*,*c*;*λ*,*λ*). *Since bc* ∈ *Fact*(*x*_○_), *Case* 2 *holds: r linearizes x*_○_ *to cab and produces* (*cab*)_○_ = {*abc*,*bca*,*cab*} = *x*_○_∙*λ*_○_.
- *Let x*_○_ = (*ab*)_○_ *and y*_○_ = (*a*)_○_, *which satisfy none of the three cases. Then x*_○_∙*y*_○_ = {*aba*,*baa*}: *the letter b*, *which does not occur in y*, *is not the last letter of any of its words, so the conjugate aab is missing and x*_○_∙*y*_○_ *is not a circular word language. Since every Păun splicing produces circular words, no Păun rule yields x*_○_∙*y*_○_*;* e.g.*,* (*λ*,*λ*;*λ*,*λ*) *produces* (*aab*)_○_ = {*aab*,*aba*,*baa*} ≠ *x*_○_∙*y*_○_.

In the next part, we characterize when the Pixton circular splicing operation reproduces the one-wheel concatenation.

**Theorem** **4.** *Let* $x_{○}={\left(x_{1}\ldots x_{n}\right)}_{○}$ *and* $y_{○}={\left(y_{1}\ldots y_{m}\right)}_{○}$ *be any arbitrary circular words over the finite alphabet* Σ *with indices taken modulo n and m respectively. Then applying Pixton’s circular splicing with rules r* = (*λ*,*λ*;*λ*) *and* $\bar{r}=(\lambda ,\lambda ;\lambda )$ (*in fact, this rule is associated with itself*) *leads to the same set as the one-wheel concatenation x*_○_⊙*y*_○_.

**Proof.**  On the one hand, in a Pixton rule *r* = (*α*,*α*′;*β*) setting both *α* and *α*′ to the empty word, the rule can be applied anywhere to cut and linearize the two circular input words. Further, by setting *β* to the empty word we do not permit changes by rewriting, and thus, the conjugates of the original circular words are concatenated, and the result is closed to be a circular word again. Thus, in this case, exactly the one-wheel concatenation is obtained. □

**Example** **11.** *Let x*_○_ = (*ab*)_○_ *and y*_○_ = (*c*)_○_*. By Corollary 1, the Păun rule* (*λ, λ; λ, λ*) *linearizes x*_○_ *to ab or ba and y*_○_ *to c, producing the two distinct circular words* (*abc*)_○_ *and* (*bac*)_○_*; this is exactly x*_○_⊙*y*_○_*. By Theorem 6, the Pixton rule pair* $r=\bar{r}=(\lambda ,\lambda ;\lambda )$ *produces the same set.*

Now, analogously to Theorem 3, we can also state a result when applying Pixton’s circular splicing results in the same set as the two-wheel concatenation of the input circular words.

**Theorem** **5.** *Let* $x_{○}={\left(x_{1}\ldots x_{n}\right)}_{○}$ *and* $y_{○}={\left(y_{1}\ldots y_{m}\right)}_{○}$ *be any arbitrary circular words over the finite alphabet* Σ *with indices taken modulo n and m respectively. The Pixton circular splicing applied to them has the same result as their two-wheel concatenation if and only if* (*at least*) *one of the following three cases holds*:

1. *x*,*y* ∈ {*a*}^*^ *for any a* ∈ Σ*, and* $r,\bar{r}$ *such that r* = (*a^p^*,*a^q^*;*β*) *and* $\bar{r}=(a^{q},a^{p};\beta \prime )$ *, where p*,*q* ∈ ℕ_0_ *with* 0 ≤ *p* ≤ |*x*|*,* 0 ≤ *q* ≤ |*y*| *and ββ*′ = *a^p^*^+*q*^*;*
2. $x_{○}={\left(x_{1}\ldots x_{n}\right)}_{○}\neq \lambda_{○}$*, y*_○_ = *λ*_○_ *and r* = (*α*,*λ*;*β*) *and* $\bar{r}=\left(\lambda ,\alpha ;\beta \prime \right)$ *such that α* ∈ *Fact*(*x*_○_) *and ββ*′ = *α;*
3. *x*_○_ = *λ*_○_*,* $y_{○}={\left(y_{1}\ldots y_{m}\right)}_{○}\neq \lambda_{○}$ *and r* = (*λ*,*α*′;*β*) *and* $\bar{r}=\left(\alpha \prime ,\lambda ;\beta \prime \right)$ *such that α*′ ∈ *Fact*(*y*_○_) *and ββ*′ = *α*′.

**Proof.**  (Necessary condition) Assume that the Pixton circular splicing operations defined by a pair ($r,\bar{r}$) applied to *x*_○_ and *y*_○_ results in *x*_○_∙*y*_○_. As this operation also produces a circular word language, Lemma 1 applies exactly as in Theorem 3, so either *x*,*y* ∈ {*a*}^*^ for some *a* ∈ Σ, or one of the two circular words is *λ*_○_.

Let *x*,*y* ∈ {*a*}^*^. Writing *r* = (*α*,*α′*;*β*) and $\bar{r}=\left(\alpha^{\prime },\alpha ;\beta^{\prime }\right)$, the applicability to *x*_○_ and *y*_○_ gives *α* = *a^p^*, *α′* = *a^q^* with 0 ≤ *p* ≤ |*x*| and 0 ≤ *q* ≤ |*y*|, and the produced circular word must be ${\left(a^{\left|x\right|+\left|y\right|}\right)}_{○}$; comparing the lengths of *εβε′β′* = *a*^|*x*|−*p*^*βa*^|*y*|−*q*^*β′* and of *a*^|*x*|+|*y*|^ yields *ββ′* = *a^p^*^+*q*^, which is alternative (1) in the theorem. This case also covers the case that both of *x*_○_ and *y*_○_ are the empty circular word.

However, if either *x*_○_ = *λ*_○_ or *y*_○_ = *λ*_○_, then the proof is immediate and yields alternatives (2) or (3). Indeed, suppose *y*_○_ = *λ*_○_. Then every conjugate *u* ∈ *x*_○_ must be obtainable as the output of the Pixton rule pair $\left(r,\;\bar{r}\right)$ applied to representatives of *x*_○_ and the empty circular word. Writing a general pair as *r* = (*α*,*α′*;*β*) with $\bar{r}=\left(\alpha^{\prime },\alpha ;\beta^{\prime }\right)$, we must have *α′* = *λ*; hence *r* = (*α*,*λ*;*β*) and $\bar{r}=\left(\lambda ,\alpha ;\beta^{\prime }\right)$. To produce *u*, we must have a decomposition *u* = *εβ* with *αε* ∈ *x*_○_; comparing this equality for the various conjugates forces *ββ′* = *α*. This is exactly alternative (2). The symmetric argument gives (3) when *x*_○_ = *λ*_○_.

(Sufficient condition) We show each alternative implies that applying the given pair of rules, the two-wheel concatenation *x*_○_∙*y*_○_ is obtained.

- If alternative (1) holds: *x* = *a*^|*x*|^, *y* = *a*^|*y*|^ and there exists *r* = (*a^p^*,*a^q^*;*β*) with partner $\bar{r}=(a^{q},a^{p};\beta \prime )$ and *ββ*′ = *a^p^*^+*q*^. Take any *u* = *a^s^* ∈ *x*_○_, *v* = *a^t^* ∈ *y*_○_. Choose *ε* = *a^s^*^−*p*^, *ε*′ = *a^t^*^−*q*^ (these are nonnegative powers because 0 ≤ *p* ≤ |*x*|*,* 0 ≤ *q* ≤ |*y*|). Then the Pixton pair applied to ${\left(a^{p}\varepsilon \right)}_{○}$ and ${\left(a^{q}\varepsilon \prime \right)}_{○}$ produces:
  *εβε′β′* = *a^s^*^−*p*^*βa*^t−*q*^*β′* = *a*^s−*p*^*a^p^*^+*q*^*a*^t−*q*^ = *a^s^*^+*t*^ = *uv*.
  Thus, every *uv* ∈ *x*_○_∙*y*_○_ is realized; conversely, any Pixton output is plainly a concatenation of a conjugate of *x*_○_ and a conjugate of *y*_○_. Hence, the equality holds.
- If alternative (2) holds: *y*_○_ = *λ*_○_ and *r* = (*α*,*λ*;*β*), $\bar{r}=\left(\lambda ,\alpha ;\beta^{\prime }\right)$ with *α* ∈ *Fact*(*x*_○_) and *ββ′* = *α*. Take any *u* = *αε* ∈ *x*_○_, then applying the Pixton pair to (*αε*)_○_ and *λ*_○_ yields:
  *εβε′β′* = *εββ′* = *εα* = *u*.
  So, every conjugate *u* of *x* is produced; the concatenation with the empty word yields exactly *x*_○_∙*y*_○_.
- The proof of alternative (3) is identical to (2). □

**Example** **12.** *Let* $x_{○}={\left(a^{2}\right)}_{○}$ *and y*_○_ = (*a*)_○_*, and take p* = *q* = 1*. The pair r* = (*a*,*a*;*a*)*,* $\bar{r}=(a,a;a)$ *satisfies ββ′* = *a^p^*^+*q*^ = *a*^2^*, so Case 1 of Theorem* 5 *holds: with ε* = *a and ε′* = *λ, it produces* ${\left(\varepsilon \beta \varepsilon^{\prime }\beta^{\prime }\right)}_{○}={\left(a^{3}\right)}_{○}=x_{○}\cdot y_{○}$*. In contrast, the pair r* = (*a*,*a*;*a*^2^)*,* $\bar{r}=(a,a;a)$ *has ββ′* = *a*^3^ ≠ *a*^2^*, and it produces* ${\left(a^{4}\right)}_{○}\neq x_{○}\cdot y_{○}$*, since Pixton’s operation may change the length.*

## 4. Computing by Circular Insertion–Deletion Operations

The insertion of linear DNA strands into linear DNA strands is modeled by contextual insertion [14]. In the circular framework, insertion–deletion operations are particularly relevant for representing transformations of circular DNA, leading to computational models grounded in circular molecular structures (and, in the theoretical description, by circular word structures).

### 4.1. Circular Insertion

In [15], the circular context-guided insertion was introduced. It was first defined as the insertion, between the factors *u* and *v* of a linear word *x*, of each of the linear words representing a circular permutation of *y*. We recall a form of the insertion operation [15]:

**Definition 6** **(Circular Insertion into Linear Word).** *Let* Σ* be a finite alphabet. A circular insertion rule is represented by a triple of the form r_ins_* = (*u*, *λ*/*β*_○_, *v*), *where u, v*∈Σ^*^ *and* *β*∈Σ^+^*. Then, given a linear word x* = *x*_1_*uvx*_2_*, the application of the rule inserts any conjugate γ of β*_○_ *between u and v, obtaining y* = *x*_1_*u*γ*vx*_2_*. We denote this operation by:*

(4) $$x{⊢}_{r_{\mathrm{ins}}}y=\left\{\;x_{1}u\gamma vx_{2}\;|\;x=x_{1}\mathrm{uv}x_{2},\;\gamma \in \beta_{○},x_{1},x_{2}\in \Sigma^{*}\;\right\}$$

**Figure 4:**
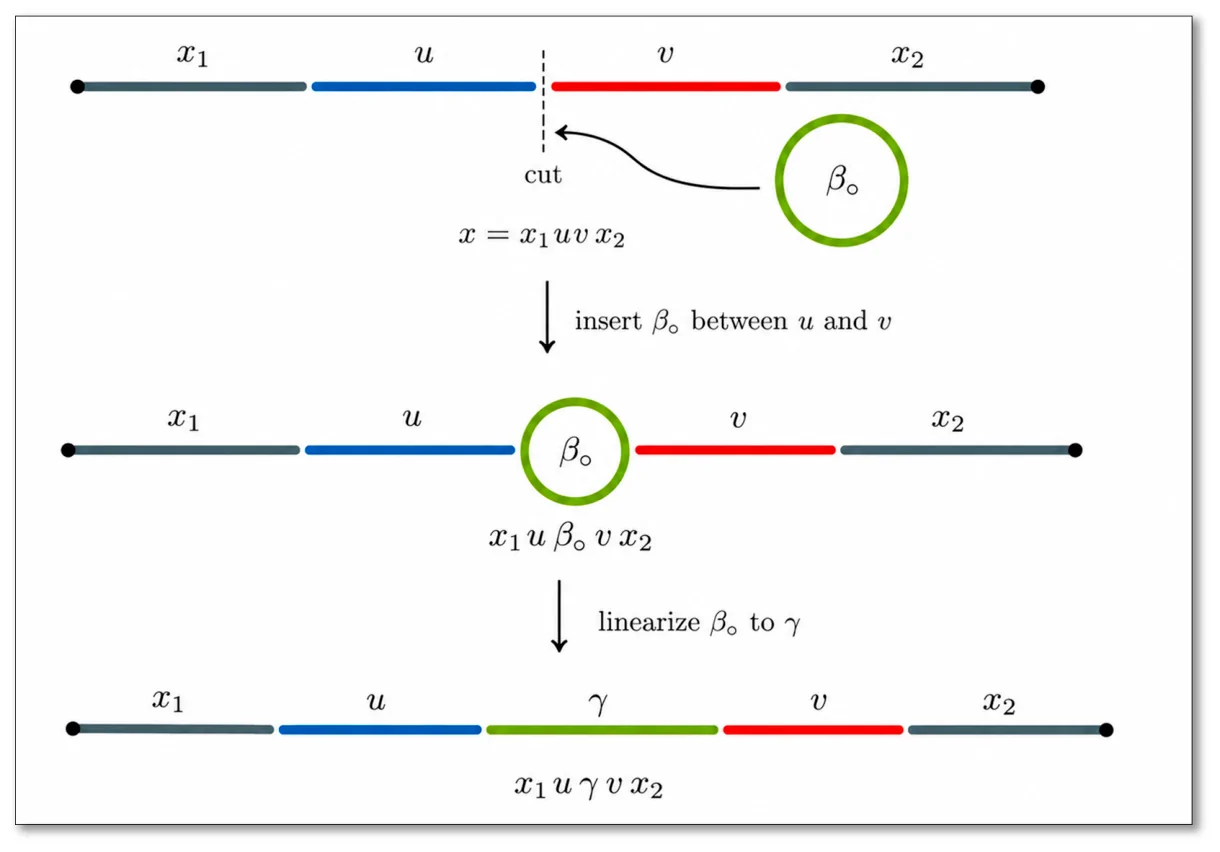
Circular Insertion into a Linear Word.

Although an application of the rule may give only one particular word, as we have already noted for the splicing operations, in general a set of possible results can be obtained, as we have written here in the definition. A graphical explanation is also shown in Figure 4. On the one hand, if *x* does not contain *uv* as a factor, then the result of the circular contextual insertion of any word into *x* is nothing, i.e., the empty set. The cardinality of the result of $x{⊢}_{r_{\mathrm{ins}}}y$ ranges from 0 to the number of occurrences of *uv* times |*β*_○_|. We note here that in some practical applications the inserted circular strands are short, and in some models only some of the conjugates can be inserted based also on the context where the cycle is broken.

**Figure 5:**
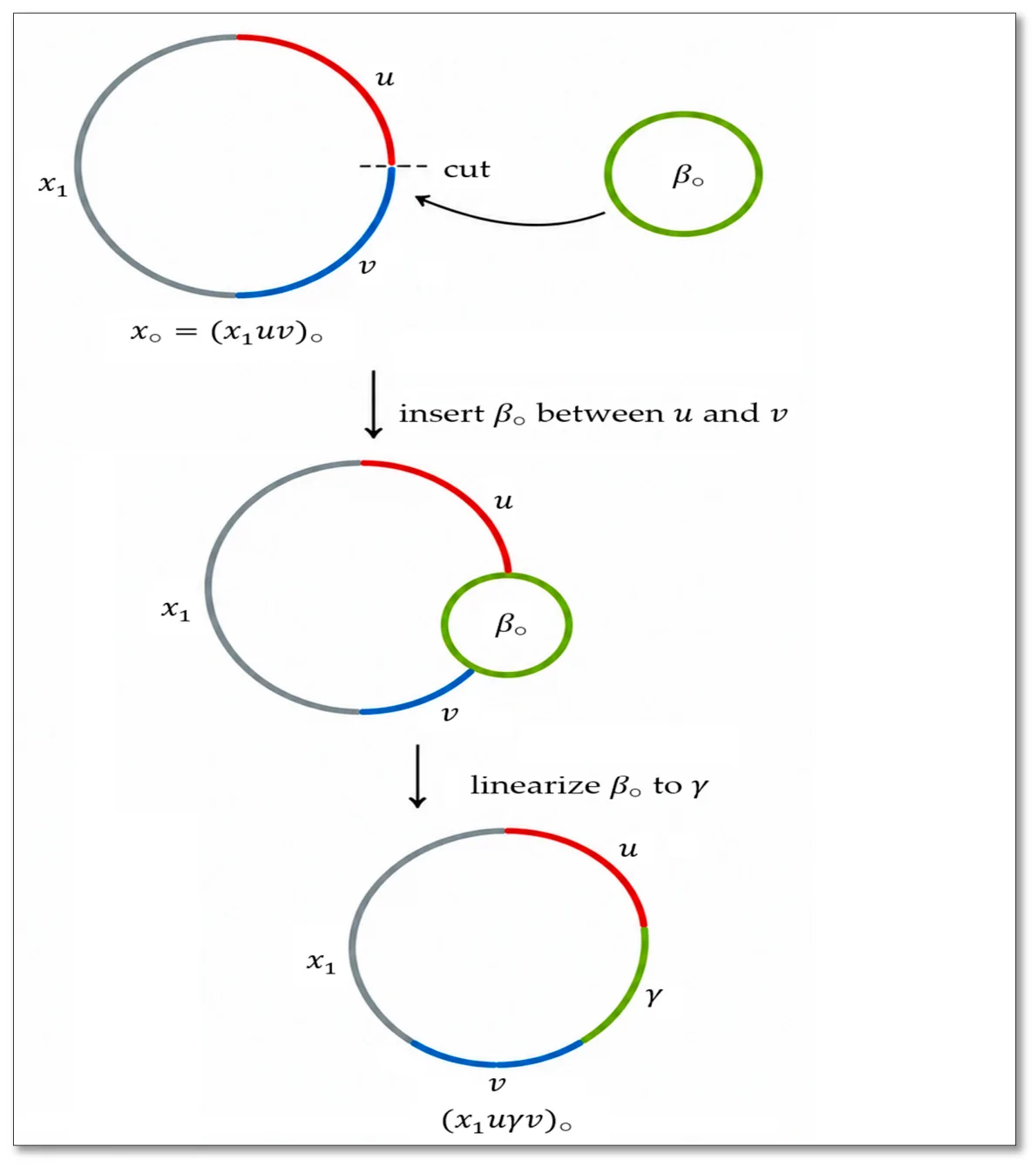
Circular Insertion into a Circular Word.

Based on the above definition, since we are interested in having circular words as results of an operation, we introduce the insertion of a circular strand of DNA into another circular strand (see Figure 5). In order to simulate this process, we examine here a generalization of word insertions.

**Definition 7** **(Circular Insertion into Circular Word).** *Let* Σ *be a finite alphabet. A circular insertion rule is represented by a triple of the form r_ins_* = *(u,λ/β*_○_*,v), where u, v* ∈ Σ^*^ *and β* ∈ Σ^+^. *Then, having a circular word* $x_{○}={\left(\mathrm{uv}x_{1}\right)}_{○}$*, the application of the rule inserts any conjugate γ of β*_○_ *between u and v, obtaining* $y_{○}={\left(u\gamma vx_{1}\right)}_{○}$*. We denote this operation by:*

(5) $$x_{○}{⊢}_{r_{\mathrm{ins}}}y_{○}=\left\{z\in {\left(u\gamma vx_{1}\right)}_{○}\;|\;x=\mathrm{uv}x_{1},\;\gamma \in \beta_{○},x_{1}\in \Sigma^{*}\right\}$$

In Definition 6, the contexts *u* and *v* are searched for in a linear word *x*, i.e., in one fixed linearization of the molecule, and the result *x*_1_*uγvx*_2_ is again a linear word (see Figure 4). In Definition 7, they are searched for in the circular word *x*_○_, i.e., in all of its linearizations at the same time, and the result is a circular word (see Figure 5). The two operations therefore differ in three respects. First, Definition 7 is closed on circular words, so it can be iterated on cyclic molecules and compared with the circular splicing operations and with the concatenations of circular words (Theorems 13 and 14), which is not possible for Definition 6. Second, Definition 7 is a conservative extension of Definition 6: by Lemma 2, it can be obtained by applying Definition 6 to all linearizations of *x*_○_ and closing the results under conjugation. Third, and most importantly, this closure is a genuine enlargement and not a reformulation: as we will show, by Lemma 3, we only have the inclusions *Z* ⊆ Z′ ⊆ Z″, and Theorem 6 characterizes when equality holds, the inclusions being strict as soon as *x*_○_ has an occurrence of the context *uv* that spans the position at which *x* was cut, i.e., an occurrence that no linear representative of the molecule exhibits (see Example 13, where *x* = *cdaab* yields nothing, while ${x_{○}=(c\mathrm{daab})}_{○}$ yields three circular words). In biological terms, a cyclic molecule has no distinguished starting point, so the recognition site of the enzyme may lie across any point of the cycle; a model based on a fixed linearization would discard exactly these occurrences.

Observe that, since it is clear from the context, we use the same notation for a circular insertion rule whether we apply it to a linear or to a cyclic word.

Now, as a preliminary result about circular insertions*,* we show that our new definition is a natural extension of Definition 6 to circular words (i.e., it makes the insertion applicable to the case when both the original words are cyclic words, not only the one that is inserted).

**Lemma** **2.** *Applying an insertion rule r_ins_* = (*u*,*λ*/*β*_○_,*v*) *to a circular word x*_○_ *yields the same result as the following process: linearize the circular word x*_○_ *in all possible ways; apply the rule r_ins_ to each linear word by inserting any conjugate γ of β*_○_ *between u and v in them (according to Definition 6); then close each resulting word back into its circular form.*

**Proof.**  The result is a direct consequence of the new definition when it is compared with the original definition applied for inserting circular words into linear words. □

**Lemma** **3.** *Let r_ins_* = (*u*,*λ*/*β*_○_,*v*) *be a circular insertion rule over the alphabet* Σ *and a linear word x be given. Let the sets Z, Z′ and Z″ represent the results of operations r_ins_ to x, the set of all conjugates of this set and the application of r_ins_ to the circular word x*_○_ *as a circular insertion to a circular word, respectively. Then, we have the following relation:*

*Z* ⊆ Z′ ⊆ Z″ (6)

**Proof.**  The first part of the proof is trivial, since Z’, as the set of all conjugates of Z, must be a superset of Z. On the other hand, each element of Z’ is a conjugate of a word obtained by inserting a conjugate *γ* of *β*_○_ between *u* and *v* in *x* (if such position exists). However, in this case, this word is also a conjugate of a linear word which is in $x_{○}{⊢}_{r_{\mathrm{ins}}}y_{○}$. □

The previous lemma shows a real hierarchy of the obtained sets. To see and prove that these sets may not be equal, we present the following examples.

**Example** **13.** *Let* Σ = {*a,b,c,d*,0,1,2} *be an alphabet, and let r_ins_* = (*ab,λ*/(012)_○_*,cd) be a circular insertion rule. Considering the linear word x = cdaab over* Σ*, the rule r_ins_ applied to x generates nothing other than the empty set since it does not contain abcd as a factor. Hence,* $x{⊢}_{r_{\mathrm{ins}}}y=\{\}$ *and* ${\left(x{⊢}_{r_{\mathrm{ins}}}y\right)}_{○}=\{\}$*. However, the rule r_ins_ applied to the circular word* ${x_{○}=(c\mathrm{daab})}_{○}$ *generates* $x_{○}{⊢}_{r_{\mathrm{ins}}}y_{○}={\left(\mathrm{ab}012\mathrm{cda}\right)}_{○}\cup {\left(\mathrm{ab}120\mathrm{cda}\right)}_{○}\cup {\left(\mathrm{ab}201\mathrm{cda}\right)}_{○}$.

*Moreover, applying the rule r_ins_ to the linear word w = abcd, it produces* $w{⊢}_{r_{\mathrm{ins}}}y=\{\mathrm{ab}012\mathrm{cd},\mathrm{ab}120\mathrm{cd},\mathrm{ab}201\mathrm{cd}$} *while the set of all its conjugates is*

$${\left(w{⊢}_{r_{\mathrm{ins}}}y\right)}_{○}=\left\{\begin{array}{c} \mathrm{ab}012\mathrm{cd},b012\mathrm{cda},012\mathrm{cdab},12\mathrm{cdab}0,2\mathrm{cdab}01,\mathrm{cdab}012,\mathrm{dab}012c\\ \mathrm{ab}120\mathrm{cd},b120\mathrm{cda},120\mathrm{cdab},20\mathrm{cdab}1,0\mathrm{cdab}12,\mathrm{cdab}120,\mathrm{dab}120c\\ \mathrm{ab}201\mathrm{cd},b201\mathrm{cda},201\mathrm{cdab},01\mathrm{cdab}2,1\mathrm{cdab}20,\mathrm{cdab}201,\mathrm{dab}201c \end{array}\right\}$$

and

$$w_{○}{⊢}_{r_{\mathrm{ins}}}y_{○}={\left(\mathrm{ab}012\mathrm{cd}\right)}_{○}\cup {\left(\mathrm{ab}120\mathrm{cd}\right)}_{○}\cup {\left(\mathrm{ab}201\mathrm{cd}\right)}_{○}$$

Now, we turn to an important result by further analyzing this second part of the relation.

**Theorem** **6.** *Let* Σ *be a finite alphabet, and r_ins_* = (*u*,*λ*/*β*_○_,*v*) *be a circular insertion rule, where u*, *v* ∈ Σ^*^ *and β* ∈ Σ^+^. *For any linear word x*,

1. *If* ${\left(x{⊢}_{r_{\mathrm{ins}}}y\right)}_{○}=x_{○}{⊢}_{r_{\mathrm{ins}}}y_{○}$*, then uv* ∈ *Fact*(*x*) *or uv* ∉ *Fact*(*x*_○_).
2. *If uv* ∉ *Fact*(*x*_○_)*, or the number of occurrences of uv in x is maximal in the sense that the suffix of x concatenated to the prefix of x does not provide any extra occurrences of uv, then* ${\left(x{⊢}_{r_{\mathrm{ins}}}y\right)}_{○}=x_{○}{⊢}_{r_{\mathrm{ins}}}y_{○}$.

**Proof.**  Case 1: Assume that ${\left(x{⊢}_{r_{\mathrm{ins}}}y\right)}_{○}=x_{○}{⊢}_{r_{\mathrm{ins}}}y_{○}$. Let us suppose that *uv* ∉ *Fact*(*x*) and *uv* ∈ *Fact*(*x*_○_). Then, ${\left(x{⊢}_{r_{\mathrm{ins}}}y\right)}_{○}$ is the empty set, whereas $x_{○}{⊢}_{r_{\mathrm{ins}}}y_{○}$ is not, which leads to a contradiction. Therefore, *uv* ∈ *Fact*(*x*) or *uv* ∉ *Fact*(*x*_○_).

Case 2: Assume that the linear word *x* has the factor *uv*, i.e., *x* can be written in the form of *x* = *x*_1_*uvx*_2_. By Lemma 3, ${\left(x{⊢}_{r_{\mathrm{ins}}}y\right)}_{○}\subseteq x_{○}{⊢}_{r_{\mathrm{ins}}}y_{○}$. Conversely, each word of $x_{○}{⊢}_{r_{\mathrm{ins}}}y_{○}$ arises from an occurrence of *uv* in *x*_○_ and some *γ* ∈ *β*_○_. If the number of occurrences of *uv* in *x* is maximal as it is described, then the result is $y_{○}={\left(u\gamma vx_{2}x_{1}\right)}_{○}$ for each conjugate of *x*_○_ having an occurrence of *uv* as a prefix, whose words belong to ${\left(x{⊢}_{r_{\mathrm{ins}}}y\right)}_{○}$. This leads to the equality ${\left(x{⊢}_{r_{\mathrm{ins}}}y\right)}_{○}=x_{○}{⊢}_{r_{\mathrm{ins}}}y_{○}$.

On the other hand, if *uv* ∉ *Fact*(*x*_○_), then both ${\left(x{⊢}_{r_{\mathrm{ins}}}y\right)}_{○}$ and $x_{○}{⊢}_{r_{\mathrm{ins}}}y_{○}$ are the empty sets. Hence, the equality holds. *□*

Notice that the condition *uv* ∈ *Fact*(*x*) itself is not sufficient for the set equality of the previous theorem since *x*_○_ can also contain occurrence(s) that run(s) across the junction between the last and first letters of *x*, as we show in the next example.

**Example** **14.** *Let r_ins_* = (*a*,*λ*/(0)_○_,*b*) *and x* = *babca. In x, the factor ab occurs only once, giving ba*0*bca. So* ${\left(x{⊢}_{r_{\mathrm{ins}}}y\right)}_{○}={\left(\mathrm{ba}0\mathrm{bca}\right)}_{○}$ *. In x*_○_*, ab also occurs across the junction (last a, first b), giving the extra circular word* (*babca*0)_○_*. Since ba*0*bca is not a conjugate of babca*0*, then* ${\left(x{⊢}_{r_{\mathrm{ins}}}y\right)}_{○}$ *and* $x_{○}{⊢}_{r_{\mathrm{ins}}}y_{○}$ *differ even though uv* ∈ *Fact*(*x*).

The following results relate the circular insertion to Păun circular splicing operations and the one-wheel concatenation.

**Theorem** **7.** *Let* Σ *be a finite alphabet, and r_ins_* = (*u*,*λ*/*β*_○_,*v*) *be a circular insertion rule, where u*, *v* ∈ Σ^*^ *and β* ∈ Σ^+^. *Then, the circular insertion by r_ins_ can be simulated by a Păun circular splicing operation.*

**Proof.**  Consider the Păun rule *r* = (*u*,*v*;*λ*,*λ*). Let $x_{○}={\left(\mathrm{uv}x_{1}\right)}_{○}$ (for some *x*_1_ ∈ Σ^*^ if any) and *y*_○_ = *β*_○_. Applying *r* to *x*_○_ and *y*_○_, the Păun operation linearizes the words to *uvx*_1_ (for any occurrence of *uv* in *x*_○_) and to any conjugate *γ* of *β*_○_, concatenates them, and circularizes the result, yielding ${\left(u\gamma vx_{1}\right)}_{○}$, whose words are exactly the results of applying the circular insertion rule *r_ins_* to *x*_○_. □

**Example** **15.** *Let r_ins_* = (*a*,*λ*/(01)_○_,*b*) *and x*_○_ = (*abc*)_○_*. The circular insertion produces* (*a*01*bc*)_○_ *and* (*a*10*bc*)_○_*. The Păun rule* (*a*,*b*;*λ*,*λ*) *linearizes x*_○_ *to bca and* (01)_○_ *to* 01 *or* 10*, producing* (*bca*01)_○_ = (*a*01*bc*)_○_ *and* (*bca*10)_○_ = (*a*10*bc*)_○_*,* i.e.*, the same set.*

**Theorem** **8.** *Let* Σ *be a finite alphabet, and r_ins_* = (*λ*,*λ*/*β*_○_,*λ*) *be a circular insertion rule, β* ∈ Σ^+^. *Consider the circular word x*_○_. *Then, the set of circular words obtained by applying the circular insertion rule r_ins_ to x*_○_ *coincides with the one-wheel concatenation x*_○_⊙*β*_○_.

**Proof.**  Let *y*_○_ be obtained from *x*_○_ by *r_ins_*. Since *λ* appears anywhere in *x* as a subword, any conjugate *x*_1_ of *x* can be taken as a linearization, i.e., any point can be used to cut the circular word *x*_○_. By the definition of circular insertion, there exists a conjugate *γ* ∈ *β*_○_ such that $y_{○}={\left(\gamma x_{1}\right)}_{○}$. Hence, *y*_○_ ⊂ *x*_○_⊙*β*_○_.

For the other inclusion, let *y* ∈ *x*_○_⊙*β*_○_. Then, there exist conjugates *x*_1_ ∈ *x*_○_ and *γ* ∈ *β*_○_ and a circular word $y_{○}^{\prime }={\left(x_{1}\gamma \right)}_{○}$ such that $y\in y_{○}^{\prime }$. Thus, actually, *y* is a conjugate of a concatenation of the form *x*_1_*γ*. Applying the insertion rule *r_ins_* to *x*_○_, as the empty word is used as context, may cut the cycle of *x*_○_ to get the conjugate *x*_1_. The insertion of *γ* ∈ *β*_○_ at this point yields exactly to ${\left(x_{1}\gamma \right)}_{○}=y_{○}^{\prime }$. Thus, as $y\in y_{○}^{\prime }$, it is obtainable by this circular insertion. This completes the second direction of the proof. □

**Example** **16.** *Let r_ins_* = (*λ*,*λ*/(01)_○_,*λ*) *and x*_○_ = (*ab*)_○_*. The circular insertion produces the four distinct circular words* (*ab*01)_○_*,* (*ab*10)_○_*,* (*ba*01)_○_ *and* (*ba*10)_○_*, which form exactly x*_○_⊙(01)_○_.

### 4.2. Circular Deletion

For the sake of completeness, we also consider the deletion operation on circular words. Symmetrically to Definition 7, we define it as follows:

**Definition 8** **(Circular Deletion from a Circular Word).** *Let* Σ *be a finite alphabet. A circular deletion rule is represented by a triple of the form r_del_* = (*u,α*_○_/*λ,v*), *where u, v* ∈Σ^*^ *and α* ∈ Σ*^+^. Then, having a circular word* $x_{○}={\left(u\gamma vx_{1}\right)}_{○}$ *with γ* ∈ *α*_○_*, the application of the rule deletes the occurrence of γ between u and v, obtaining* $y_{○}={\left(\mathrm{uv}x_{1}\right)}_{○}$*. We denote this operation by:*

(7) $$x_{○}{⊢}_{r_{\mathrm{del}}}y_{○}=\left\{z\in {\left(\mathrm{uv}x_{1}\right)}_{○}\;|\;x=u\gamma vx_{1},\;\gamma \in \alpha_{○},x_{1}\in \Sigma^{*}\right\}$$

As in the case of insertion, the result may be the empty set (if no conjugate of *x* contains *uγv* as a factor for any *γ* ∈ *α*_○_), and it may also contain the circular words obtained from several occurrences. We first make precise why this operation cannot be equivalent to any of the concatenation operations.

Both concatenation operations are length-increasing, whereas the deletion operation is strictly length-decreasing. Hence, no equivalence of the type given in Theorem 2, Corollary 1 and Theorems 4 and 8 can hold for deletion. Nevertheless, the deletion operation is closely related to the one-wheel concatenation, namely, as its inverse relation, as the next result shows.

**Theorem** **9.** *Let* Σ *be a finite alphabet, and r_del_* = (*λ*,*α*_○_/*λ*,*λ*) *be a circular deletion rule, α* ∈ Σ^+^. *Then, for any circular words x*_○_ *and y*_○_*,*

(8) $$x_{○}{⊢}_{r_{\mathrm{del}}}y_{○}\;\mathrm{if}\;\mathrm{and}\;\mathrm{only}\;\mathrm{if}\;x\in y_{○}⊙\alpha_{○}$$

**Proof.**  Assume first that *y*_○_ is obtained from *x*_○_ by *r_del_*. Since the contexts are empty, by Definition 8 there are a conjugate *γ* ∈ *α*_○_ and a word *x*_1_ ∈ Σ^*^ such that $x_{○}={\left(\gamma x_{1}\right)}_{○}$ and $y_{○}={\left(x_{1}\right)}_{○}$. As *x*_1_ ∈ *y*_○_ and *γ* ∈ *α*_○_, the word *x*_1_*γ* belongs to the set described in Definition 5, and *x*, being a conjugate of *γx*_1_, is also a conjugate of *x*_1_*γ*. Hence *x* ∈ *y*_○_⊙*α*_○_.

Conversely, assume that *x* ∈ *y*_○_⊙*α*_○_. By Definition 5, *x* is a conjugate of a word of the form *y*_1_*γ* with *y*_1_ ∈ *y*_○_ and *γ* ∈ *α*_○_. Therefore $x_{○}={\left(y_{1}\gamma \right)}_{○}={\left(\gamma y_{1}\right)}_{○}$, i.e., *x*_○_ has an occurrence of the conjugate *γ* of *α*_○_. As the contexts of *r_del_* are empty, the rule can be applied at this occurrence, and it deletes *γ*, yielding ${\left(y_{1}\right)}_{○}=y_{○}$. □

Theorem 9 is the exact counterpart of Theorem 8: while the circular insertion with empty contexts realizes the one-wheel concatenation, the circular deletion with empty contexts realizes its inverse relation, i.e., a kind of one-wheel quotient by *α*_○_. Finally, we show that circular deletion can also be related to the splicing operations.

**Theorem** **10.** *Let* Σ *be a finite alphabet, and r_del_* = (*u,α*_○_*/λ,v*) *be a circular deletion rule, α* ∈ Σ^+^. *Then, for each γ* ∈ *α*_○_*, the Pixton circular splicing operation defined by the rule pair r = (uγv,λ;uv) and* $\bar{r}=\left(\lambda ,u\gamma v;\lambda \right)$, *applied to x*_○_ *and to λ*_○_*, produces exactly those circular words that are obtained by r_del_ applying it to x_○_ using the occurrences of uγv. Hence, the finite set of rule pairs* $\left(r,\;\bar{r}\right)$ *simulates r_del_.*

**Proof.**  Let *γ* ∈ *α*_○_ be fixed. In the notation of Definition 3, the rule pair *r* = (*α*,*α′*;*β*) with *α* = *uγv*, *α′* = *λ*, *β* = *uv* and $\bar{r}=(\alpha^{\prime },\alpha ;\beta \prime )$ with *β*′ = *λ* is applicable to *x*_○_ and *λ*_○_ if and only if *x*_○_ = (*uγvε*)_○_ for some *ε* ∈ Σ^*^, i.e., if and only if *x* has a conjugate of the form *uγvε*; the second circular word is linearized as *ε′* = *λ*. In this case, the operation linearizes *x*_○_ to *εuγv*, replaces the suffix *uγv* by *β* = *uv* and the suffix *α′* = *λ* by *β*′ = *λ*, concatenates the obtained words and circularizes the result, producing (*εuv*)_○_ = (*uvε*)_○_.

On the other hand, by Definition 8, the rule *r_del_* is applicable to *x*_○_ at an occurrence of *uγv* if and only if *x*_○_ = (*uγvε*)_○_ for some *ε* ∈ Σ^*^ (writing *ε* for *x*_1_), and then it produces (*uvε*)_○_. Thus, the applications of the pair $\left(r,\;\bar{r}\right)$ to *x*_○_ and *λ*_○_ and the applications of *r_del_* at the occurrences of *uγv* correspond to each other and give the same circular words. Taking the union for all *γ* ∈ *α*_○_, which is a finite set, the whole result of *r_del_* is obtained. □

Note that similar results as Theorem 10 cannot be stated for the Head or the Păun circular splicing operations: as we have already recalled, these operations are length-increasing, that is, actually, the length of the result equals the sum of the lengths of the two input circular words, and therefore they can never produce a shorter circular word. In contrast, Pixton’s definition allows rewriting, which makes the simulation of deletion possible.

**Example** **17.** *Let* Σ = {*a*,*b*,0,1}*, r_ins_* = (*λ*,(01)_○_/*λ*,*λ*) *and x*_○_ = (*ab*01)_○_. *The only occurrence of a conjugate of* (01)_○_ *in x*_○_ *is* 01, *hence* $x_{○}{⊢}_{r_{\mathrm{del}}}y_{○}={\left(\mathrm{ab}\right)}_{○}$*. Accordingly, x* ∈ (*ab*)_○_⊙(01)_○_*, in agreement with Theorem* 9. *Further, by Theorem* 10, *the Pixton rule pair r* = (01,*λ*;*λ*), $\bar{r}=(\lambda ,01;\lambda )$ *applied to x*_○_ *and λ*_○_ *linearizes x*_○_ *to ab*01*, replaces the suffix* 01 *by λ*, *and produces* (*ab*)_○_, i.e.*, the same circular word*.

## 5. Implications for Language Generation

The results of the previous sections concern single applications of the operations. Since the operations are used in systems that generate circular languages by iterating them, we indicate here what our equivalences imply at that level. Throughout, a circular language *L* is called regular if its linearization, i.e., the set of all the linear words belonging to its circular words, is a regular language.

**Corollary** **2.** *Let L*_1_*and L*_2_*be regular circular languages over* Σ*, and let r* = (*u*_1_, *u*_2_; *u*_3_, *u*_4_) *be a Păun circular splicing rule. Then the set of circular words obtained by applying r to the pairs of L*_1_ × *L*_2_ *is a regular circular language, and it is contained in L*_1_⊙*L*_2_ *which is itself regular and is obtained for r* = (*λ*,*λ*;*λ*,*λ*).

**Proof.**  Let *Lin*(*L*_1_) and *Lin*(*L*_2_) denote the linearizations, which are regular by assumption. By Definition 2, the rule *r* uses exactly the linearizations belonging to *Lin*(*L*_1_) ∩ *u*_2_Σ^*^u_1_ and to *Lin*(*L*_2_) ∩ *u*_4_Σ^*^u_3_, which are regular, and it concatenates them, so the linear words obtained produce a regular language; its closure under conjugation is regular as well, i.e., the obtained set is a regular circular language. The inclusion in *L*_1_⊙*L*_2_ and the equality for the rule with empty contexts are the two parts of Theorem 2, applied to each pair of circular words, while the regularity of *L*_1_⊙*L*_2_ was proved in [17]. □

Thus, one application of a Păun circular splicing rule (and hence, by Proposition 1, of the corresponding Head rule) cannot leave the class of regular circular languages, and, by Corollary 1, the largest set it can produce, *L*_1_⊙*L*_2_, is already reached by a single rule with empty contexts. Consequently, the generative power of the corresponding systems does not come from the operation itself, but from the iteration and from the restriction imposed by the sites: the contexts select, at each step, a subset of a set that is fixed in advance by the inputs.

At the level of single operations, Theorems 7 and 10 relate insertion and deletion to splicing: one application of a circular insertion rule can be performed by one application of a Păun circular splicing rule, provided that the inserted circular word is available as an input, and one application of a circular deletion rule can be performed by Pixton circular splicing rule pairs applied together with the empty circular word *λ*_○_. Whether, and how, these relations extend to the languages obtained by iterating the operations is not addressed here; for insertion, this is the first of the tasks listed in the Conclusions. We note also that the operations behave differently with respect to length, and hence with respect to the descriptional resources needed: Head’s and Păun’s operations are length-increasing, so iterating them on a finite set of initial circular words yields only circular words whose lengths are sums of the lengths of the initial ones, while Pixton’s operation permits rewriting and, by Theorem 10, deletion, so that this restriction disappears.

Finally, our equivalences have a descriptional reading. By Proposition 1, the translations between Head triples and Păun rules change the size of a rule only by the length of the common middle word x, so the two formalisms are equally succinct up to an additive term. By Theorem 10, one circular deletion rule is simulated by at most ‖*α*_○_‖ ≤ |*α*| Pixton rule pairs, a linear increase whose optimality is open. In the opposite direction, Theorem 1 shows that nine different rules may have exactly the same effect, so the sets of rules describing a given operation are far from unique, and the most succinct description, a single rule with empty contexts, is precisely the one producing the largest set of circular words.

## 6. Conclusions

The results of this paper give an algebraic reading of the biologically inspired operations defined on circular words. When the contexts prescribed by the rules are empty, circular splicing and circular insertion degenerate to a purely algebraic operation, the one-wheel concatenation of circular words; when they are not empty, they only select which linearizations of the input circular words, i.e., which cut points of the cyclic molecules, may be used, and the results obtained in this way always belong to the same one-wheel concatenation. This dependence on the contexts is generally monotone: prescribing longer sites yields fewer products, the rule with empty contexts being the maximum of this ordering and the union over all rules recovering the whole concatenation. In this sense, the one-wheel concatenation is the common algebraic core of all the operations considered here, and the contexts of the rules carry their whole computational content. The coincidence with the two-wheel concatenation is, in contrast, exceptional: it occurs only in the degenerate unary and empty cases.

As single operations, those of Head and of Păun are equivalent, and both are length-increasing; Pixton’s definition, which permits rewriting, is the only one that is able to shorten the input, and it is therefore also the only one that is able to simulate deletions. Together with our generalization of the insertion and of the deletion operations to act on circular words and to produce circular words, this shows that insertion and deletion are not independent of splicing at the level of single operations: insertion is the contextual form of the one-wheel concatenation, while deletion is its inverse relation. One application of a Păun circular splicing rule to regular circular languages yields a regular circular language; the corresponding questions for the other operations are left open. We believe that our results help to study also iterated applications of the mentioned operations.

These operations have recognizable biological counterparts, and it is worth stating how far the correspondence goes. The insertion of a circular word into a circular word models the integration of a cyclic molecule, such as a plasmid, a circular viral genome or a circular transposition intermediate, into another cyclic molecule, while the deletion models the reverse process, the excision of such a segment; that deletion is the inverse relation of the one-wheel concatenation and is the formal counterpart of the integration–excision duality. In this picture, the contexts *u* and *v* of a rule represent the sites at which the enzymes act, and they are the realistic part of the model, since site-specific recombination and the action of restriction enzymes are determined precisely by such sites.

The limitations of the model should also be stressed. As usual in this branch of formal language theory, a molecule is represented by a word over a finite alphabet, so the double-stranded structure, the Watson–Crick complementarity and the orientation of the strands are not fully represented, and neither are the topological properties of cyclic DNA, such as supercoiling, knotting or catenation, which are known to influence recombination. Moreover, the operations are non-deterministic, and their results are the sets of all products that a rule may yield, whereas in a real reaction the products obtained depend on concentrations, kinetics and reversibility. Within these limits, the results have a practical reading for DNA computing and synthetic biology, where circular molecules are used as carriers of the computation and where standard assembly methods yield circular products: the one-wheel concatenation describes the complete library of circular molecules that a given pair of inputs may produce, and our characterizations delimit which of these products can be enforced by a suitable choice of the sites, which is exactly what has to be designed in a plasmid-based experiment.

Our results concern single applications of the operations, and the natural continuation is to pass to the systems built on them. We list the specific tasks we intend to address:

(1) Define a circular insertion system as a triple (Σ, *A*, *R*), where *A* is a finite set of axiom circular words and *R* is a finite set of circular insertion rules, and compare the family of circular languages generated by such systems with the family generated by Păun circular splicing systems with finite sets of axioms and rules. Theorem 7 gives one inclusion for a single step, provided that the inserted circular words belong to the axioms; the question is whether the inclusion is strict for the generated families.
(2) Determine the sizes for which circular insertion–deletion systems are computationally complete, i.e., the tuples of parameters bounding the lengths of the inserted and deleted words and of their left and right contexts, in analogy with the known results obtained for insertion–deletion systems on linear words [13,20].
(3) Decide, for given circular words *x*_○_ and *y*_○_ and a given *α* ∈ Σ^+^, whether *y*_○_ can be obtained from *x*_○_ by iterated applications of the circular deletion rule (*λ*,*α*_○_/*λ*,*λ*), and determine the complexity of this reachability problem for the one-wheel quotient; the corresponding problem for iterated circular insertion is the reachability problem for the one-wheel concatenation.
(4) Determine the descriptional complexity of the simulation given in Theorem 10: it uses one Pixton rule pair for each conjugate of *α*_○_, and the question is the minimal number of rule pairs needed to simulate one circular deletion rule, in particular, whether a single pair is always sufficient.
(5) Define an excision operation that cuts one circular word at two sites into two circular words, i.e., a splitting counterpart of the one-wheel concatenation modeling the excision of a cyclic molecule from another one, and decide whether adding it to circular splicing systems strictly increases their generative power.

Beyond these tasks, the classical problems concerning finite circular splicing systems remain open, in particular the characterization of the circular languages, especially the regular ones, generated by them, which is known only for restricted classes of systems, and the comparison of the systems based on the definitions of Head, Păun and Pixton.

## Data Availability

The original contributions presented in this study are included in the article. Further inquiries can be directed to the corresponding authors.
